# N-Acetyl Cysteine, Selenium, and Ascorbic Acid Rescue Diabetic Cardiac Hypertrophy via Mitochondrial-Associated Redox Regulators

**DOI:** 10.3390/molecules26237285

**Published:** 2021-11-30

**Authors:** Iram Mushtaq, Zainab Bashir, Mehvish Sarwar, Maria Arshad, Ayesha Ishtiaq, Wajiha Khan, Uzma Khan, Sobia Tabassum, Tahir Ali, Tahzeeb Fatima, Hadi Valadi, Muhammad Nawaz, Iram Murtaza

**Affiliations:** 1 Signal Transduction Laboratory, Department of Biochemistry, Faculty of Biological Sciences, Quaid-i-Azam University, Islamabad 45320, Pakistan; irammushtaq@bs.qau.edu.pk (I.M.); zainabbashir23@gmail.com (Z.B.); mehwishmalik609@gmail.com (M.S.); m.arshad@bs.qau.edu.pk (M.A.); ayesha@bs.qau.edu.pk (A.I.); tahirali.bch@gmail.com (T.A.); 2 Department of Biotechnology, COMSATS University Islamabad, Abbottabad Campus, Abbotabad 22060, Pakistan; wajihak@cuiatd.edu.pk; 3 Faculty of Biological Sciences, Hazara University, Mansehra 21040, Pakistan; uzmaqau2003@yahoo.com; 4 Department of Bioinformatics and Biotechnology, Islamic International University Islamabad (IIUI), Islamabad 44000, Pakistan; sobia.tabasum@iiu.edu.pk; 5 Department of Rheumatology and Inflammation Research, Institute of Medicine, Sahlgrenska Academy, University of Gothenburg, 413 46 Gothenburg, Sweden; tahzeeb.fatima@gu.se (T.F.); hadi.valadi@gu.se (H.V.)

**Keywords:** mitochondrial stress markers, reactive oxygen species, diabetes linked cardiac hypertrophy

## Abstract

Metabolic disorders often lead to cardiac complications. Metabolic deregulations during diabetic conditions are linked to mitochondrial dysfunctions, which are the key contributing factors in cardiac hypertrophy. However, the underlying mechanisms involved in diabetes-induced cardiac hypertrophy are poorly understood. In the current study, we initially established a diabetic rat model by alloxan-administration, which was validated by peripheral glucose measurement. Diabetic rats displayed myocardial stiffness and fibrosis, changes in heart weight/body weight, heart weight/tibia length ratios, and enhanced size of myocytes, which altogether demonstrated the establishment of diabetic cardiac hypertrophy (DCH). Furthermore, we examined the expression of genes associated with mitochondrial signaling impairment. Our data show that the expression of PGC-1α, cytochrome c, MFN-2, and Drp-1 was deregulated. Mitochondrial-signaling impairment was further validated by redox-system dysregulation, which showed a significant increase in ROS and thiobarbituric acid reactive substances, both in serum and heart tissue, whereas the superoxide dismutase, catalase, and glutathione levels were decreased. Additionally, the expression levels of pro-apoptotic gene PUMA and stress marker GATA-4 genes were elevated, whereas ARC, PPARα, and Bcl-2 expression levels were decreased in the heart tissues of diabetic rats. Importantly, these alloxan-induced impairments were rescued by N-acetyl cysteine, ascorbic acid, and selenium treatment. This was demonstrated by the amelioration of myocardial stiffness, fibrosis, mitochondrial gene expression, lipid profile, restoration of myocyte size, reduced oxidative stress, and the activation of enzymes associated with antioxidant activities. Altogether, these data indicate that the improvement of mitochondrial dysfunction by protective agents such as N-acetyl cysteine, selenium, and ascorbic acid could rescue diabetes-associated cardiac complications, including DCH.

## 1. Introduction

Metabolic disorders, such as those caused by high glucose levels (hyperglycemia) and abnormal lipid profiles (dyslipidemia) are associated with a dysfunctional endocrine system, clinically related to diabetes mellitus and cardiac hypertrophy [1]. In addition to metabolic aberrations and the abnormal transport of fatty acids, the pathogenesis and progression of diabetes-linked cardiac hypertrophy are also characterized by aberrant insulin signaling, ion homeostasis, autonomic dysfunction, and interstitial fibrosis [2]. These abnormalities may ultimately lead to morphological, structural, and functional impairments in the heart [3]. An excessive uptake of extracellular glucose into cardiomyocytes, and the accumulation of glycogen and lipid droplets, are hallmarks of the diabetic heart. Normal ventricular function can be altered by advanced glycation end products (AGEs), which may result in the cross-linking of collagen either by receptor-mediated release of pro-inflammatory cytokines from macrophages or by non-receptor mediated impaired nitric oxide signaling [4]. This may cause elevated oxidative stress and an unusual increase in mass in the thickening of the left ventricle, commonly known as hypertrophy [5].

It has been reported that metabolic dysregulation during diabetic conditions may cause oxidative stress, mitochondrial dysfunction, and the induction of apoptosis. Mitochondrial dysfunction can also occur due to hyperglycemia during diabetic conditions. The elevated oxidative damage in cardiomyocytes and progressive stage endothelial cell apoptosis may activate the renin-angiotensin II system, which is associated with cardiac hypertrophy, accompanied by interstitial fibrosis [6]. Some other factors responsible for cardiac complications may include the activation of different transcriptional pathways that regulate the myocardial substrate utilization. For example, peroxisome proliferator-activated receptor (PPAR) and peroxisome proliferator-activated receptor-gamma coactivator (PGC), which constitutes the PPAR/PGC-1 signaling network, and effects the mitochondrial bioenergetics and the regulation of antioxidative defense system [7].

The expression of PGC-1 α in cardiac muscles is critical due to its ability of maintaining a balance between fatty acid oxidation and mitochondrial bioenergetics [8]. The PPAR is thought to regulate ATP production and the expression of genes involved in fatty acid metabolism. More specifically, it regulates levels of triglycerides, cholesterol, very high-density lipoproteins (VHDL), and high-density lipoproteins (HDL) [9]. As such, the PPAR is considered a molecular target of a fibrate class of drugs and is an important candidate to study cardiovascular risk factors associated with cardiac pathologies. Excessive productions of reactive oxygen species (ROS) damage the structure of mitochondria and destabilize the membrane permeability, which consequently induces apoptosis [10,11,12,13]. NADPH oxidases (NOX), xanthine oxidase, nitric oxide synthase (NOS) and mitochondria are the primary biological sources of ROS production. An increased production of ROS may cause cardiac pathologies [14]. It has been demonstrated that the scavenging of oxygen free radicals and suppression of ROS formation can be achieved by natural and synthetic antioxidants [15].

Different types of antioxidant therapies are used for the treatment of cardiovascular diseases. For instance, it has been reported that N-acetyl cysteine inhibits oxidative stress, enhances cardiac adiponectin, reduces myocardial reperfusion, and protects the diabetic heart against impaired glucose utilization and the risk of myocardial infarction/injury in diabetic rats [16,17,18,19,20,21]. N-acetyl cysteine has also been shown to prevent hyperglycemia-mediated myocardial hypertrophy, and myocyte apoptosis in diabetic rats [22,23]. N-acetyl cysteine interacts with ROS and removes them from the cardiovascular system, thus facilitating a decline in cardiac fibrosis [24,25]. Ascorbic acid has also been suggested to be a potent antioxidant that improves cardiorespiratory health and diastolic function in type II diabetes (T2D) patients. However, the role of ascorbate in preventing cardiac pathology is not fully known [26,27]. Selenium acts as a free radical scavenger, but is also thought to regulate thyroid hormone synthesis, DNA synthesis, reproduction, and fertility in humans [28,29,30,31].

It has been shown that the apoptosis repressor caspase recruitment domain (ARC) and mitofusin-2 (MFN-2) are involved in mitochondrial fusion, whereas the dynamin-related protein 1 (Drp1), p53 upregulated modulator of apoptosis (PUMA) and mitochondrial fission 1 protein (FIS1) are involved in mitochondrial fission [32]. The balance in the mitochondrial fission/fusion cycle is crucial to prevent mitochondrial dysfunction. Additionally, PUMA is a pro-apoptotic gene, which interacts with the anti-apoptotic Bcl-2 protein family members and liberates Bax and Bak. In addition to PUMA and Drp1, cytochrome c is considered one of the key regulators of mitochondrial functioning, [33,34]. The cytochrome c protein is localized in the mitochondrial intermembrane space under normal physiological conditions. However, the release of cytochrome c from mitochondria to the cytosol causes the activation of the caspase family of proteases–a primary trigger for the onset of apoptosis [35]. Measuring the levels of cytochrome c leakage from mitochondria to cytosol and their release out of the cell to the culture medium or biological fluids can help to identify the degree of apoptosis [36,37,38].

Previous studies reported cardiac hypertrophy in type 1 diabetes in relation to coronary heart disease and evaluated the associated cardiovascular risk factors [39], however, the role of the above-mentioned, major mitochondrial redox signaling regulators in diabetes-induced cardiac hypertrophy are still unknown.

The present study investigates alloxan-induced diabetic cardiac hypertrophy in an in vivo model. Alloxan monohydrate is a urea derivative (a glucose analogue), which is often found to be cytotoxic through its generation of ROS, and is thought to demolish β cells of the pancreas [40]. This glucose analogue has been used to induce diabetes-associated cardiac hypertrophy in experimental animal models [40,41,42]. We examined the characteristics of cardiac hypertrophy, such as myocardial stiffness, changes in interstitial spaces and fibrosis, heart weight/body weight and heart weight/tibia length ratios, blood glucose level, serum calcium levels, cardiac-cell size and conducted a histopathological examination of heart tissue. Expression analyses of hypertrophic marker GATA-4, mitochondrial apoptotic markers Drp1, PUMA, and cytochrome c, and anti-apoptotic marker Bcl-2, MFN-2 and ARC, and fatty acid oxidation markers PGC-1α, and PPARα were also performed.

Additionally, we evaluated the regulation of antioxidant enzymes such as superoxide dismutase, catalase, and glutathione reductase, which are considered a border line of defense in metabolic pathways. The protective effects of potent antioxidants were also evaluated against oxidative damage and pathological indicators. Our data show several dysregulations in the diabetic heart, including abnormal morphology, an abnormal expression of anti-apoptotic molecules, and mitochondrial oxidative stress-associated molecules, as well as a deregulation in anti-oxidative defense enzymes and changes in the lipid profile. Our data indicate that the improvement of mitochondrial dysfunction by protective agents such as N-acetyl cysteine, selenium, and ascorbic acid could rescue diabetes-associated cardiac complications, including diabetic cardiac hypertrophy.

## 2. Results

### 2.1. Body Weights and Effects of Antioxidants

**Figure 1:**
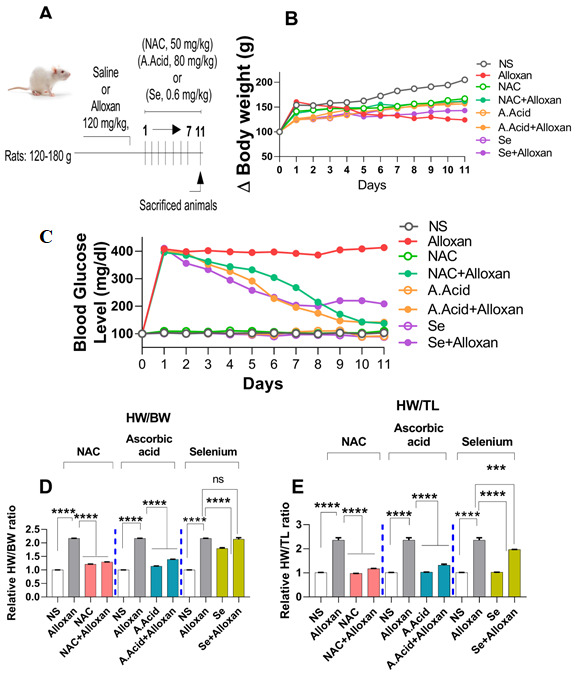
Body weight changes and morphological parameters in diabetes-induced cardiac hypertrophy. (**A**) Dosing scheme (**B**) Effect of N-acetyl cysteine, ascorbic acid, and selenium on animal body weight. The representative graph of change in body weight in experimental groups (n = 6) are shown. (**C**) Measurement of relative glucose levels (n = 6). (**D**) Cardiac hypertrophy assessment by measuring relative heart weight/body weight ratio. (**E**) Cardiac hypertrophy assessment by measuring heart weight/tibia length ratio (n = 6). NS: Normal Saline, NAC: N-acetyl cysteine, A. Acid: Ascorbic acid, Se: Selenium. Data are presented as mean ± SE of n = 6 biological samples. Significant differences are shown as *p*-values. Statistical analysis was performed using one-way ANOVA followed by Tukey’s test. (***): *p* < 0.001, (****): *p* < 0.0001.

Body weight changes are associated with hyperglycemia and an impaired glucose metabolism [42]. Therefore, we examined the initial and final body weights of experimental groups. The dosing scheme is presented in Figure 1A. Results show that alloxan induction significantly decreased the final body weight from day 1 to 11. In contrast, the treatment with antioxidants such as N-acetyl cysteine (alloxan+N-acetyl cysteine), ascorbic acid (alloxan+ascorbic acid), and selenium (alloxan+selenium) led the experimental group to regain body weight progressively from day 1 to day 11 (Figure 1B, and Supplementary Figure S1).

### 2.2. Changes in Blood Glucose Levels and Morphology in Diabetes Induced Cardiac Hypertrophy Model

#### Blood Glucose Levels and Effects of N-acetyl Cysteine

The maintenance of glucose homeostasis is an essential factor for the proper functioning of metabolic pathways in the body. Impaired glucose homeostasis can lead to a condition known as hyperglycemia, which causes cardiac complications [40]. The administration of glucose analogue alloxan induces the destruction of beta cells and produces insulinopenia, which causes Type 1 diabetes [41].

Blood glucose levels were examined in an alloxan-induced model of diabetogenic cardiac pathology. Our results showed that glucose levels were significantly increased in the blood of diseased (alloxan) group from day 1 to day 11 (Figure 1C). However, when alloxan groups were treated with antioxidants such as N-acetyl cysteine (alloxan+N-acetyl cysteine), ascorbic acid (alloxan+ascorbic acid), and selenium (alloxan+selenium), the glucose levels were significantly reduced. The data show that there was a significant change in initial and final glucose levels in the experimental groups (Figure 1C, and Supplementary Figure S2). N-acetyl cysteine and ascorbic acid conferred a better antidiabetic potential in comparison to selenium.

Furthermore, certain baseline characteristics such as ratios between heart weight/body weight, and heart weight/tibia length (hallmarks of cardiac hypertrophy) were evaluated, and were found to be significantly increased in diseased subjects. However, when treated with antioxidants, a significant reduction in these ratios was observed in disease (alloxan) groups. The N-acetyl cysteine treatment (alloxan+N-acetyl cysteine) and ascorbic acid treatment (alloxan+ascorbic acid) showed a more pronounced effect on the heart weight/body weight ratio, and relative heart weight/tibia length ratio, as compared to selenium (alloxan+selenium) (Figure 1D,E)**.**

### 2.3. Cellular Organization, Structural Changes, and Tissue Damage in Diabetes-Induced Cardiac Pathology Model

**Figure 2:**
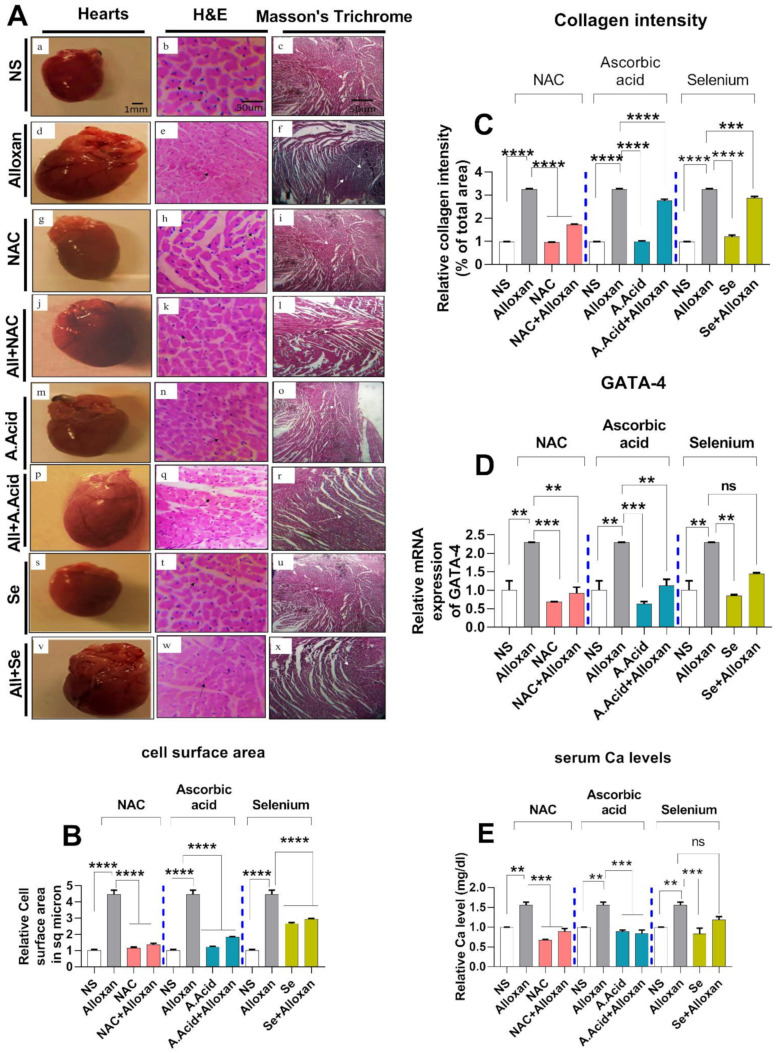
Protective effects of N-acetyl cysteine, ascorbic acid, and selenium in Diabetes induced cardiac hypertrophy. (**A**) Representation of morphological images of hearts. Corresponding sections of cardiac tissues presented by Hematoxylin and Eosin (H & E) and Masson’s Trichrome staining are shown. NS (a,b,c), Alloxan (d,e,f), NAC (g,h,i), All+NAC (j,k,l), A.Acid (m,n,o), All+A.Acid (p,q,r), Se (s,t,u), All+Se (v,w,x). NS: Normal Saline, All: Alloxan, NAC: N-acetyl cysteine All+NAC: Alloxan+N-acetyl cysteine A.Acid: Ascorbic acid All+A.Acid: Alloxan+Ascorbic acid Se: Selenium All+Se: Alloxan+Selenium, scale bar = 1mm, scale bar = 50 µm, respectively, In H & E staining, black arrows indicate cell size which is measured by Spotcam W Software (computer-assisted modeling). In Masson’s Trichrome staining panel, the white arrows indicate an area with fibrosis. (**B**) Graphical representation of cross-sectional cell size examination by SpotCam W software. (**C**) Graphical representation of collagen deposition in rat heart tissues by MIPAR^TM^.2017 software. (**D**) Relative mRNA expression of GATA 4 (n = 6) GAPDH was used as an internal control. (**E**) Analysis of serum calcium levels in pathology model (n = 6). Data are presented as mean ± SE of n biological samples. Significant differences are shown as *p*-values. Statistical analysis was performed using one-way ANOVA followed by Tukey’s test. (**): *p* < 0.01), (***): *p* < 0.001, (****): *p* < 0.0001.

Since diabetic-linked cardiac pathology represents the perturbation in cellular organization, structural changes, and tissue damage, we assessed myocardial stiffness, changes in interstitial spaces, fibrosis by Hematoxylin and Eosin staining, as well as Masson’s trichrome staining in the diabetic experimental rat model (Figure 2A). First, the cell size was measured by (SpotCam W software), which is the marker of cardiac hypertrophy. In total, 100-200 cardiomyocytes were examined in 20–50 fields. Our results show that the cell size increased in alloxan group (Figure 2B). A hematoxylin and Eosin histopathological analysis of the disease group showed an increase in cardiomyocyte size, cell shrinkage, and disturbed nucleus to cytoplasm ratio, fragmented nuclei, and a distortion in the cardiac myofibrils (Figure 2A(e)). However, in the control group (saline), the nucleus to cytoplasm ratio was found to be normal, the nuclei were single, prominent, and centrally located (Figure 2A(b)).

####  N-acetyl Cysteine Treatment Restores Myocardial Integrity

After confirming that cardiomyocyte integrity is hampered under diabetic conditions, we further examined whether N-acetyl cysteine (antioxidant) treatment could impart cardiac-protective effects against diabetic conditions. Our data reveal that antioxidant treatment recovered the myocardial integrity, and cellular organization. A histopathological examination revealed that the antioxidant treatment groups recovered the normal cell size and surface area in comparison to the disease group (Figure 2A(k,q,w)). Additionally, the N-acetyl cysteine, ascorbic acid and selenium-treated groups resumed normal cellular architecture (Figure 2A(h,n,t)). N-acetyl cysteine treatment (alloxan+N-acetyl cysteine) led to a more protective effect than ascorbic acid (alloxan+ascorbic acid) and selenium (alloxan+selenium) treatment (Figure 2A). Collectively, the data indicate that glucose-induced oxidative damage to the cell and structural proteins may cause disorientation in the cellular organization in the alloxan-induced diabetogenic model, and depict myocardial injury, which can be recovered by N-acetyl cysteine.

### 2.4. Cardiac Fibrosis in Diabetes-Induced Cardiac Hypertrophy Model

To confirm cardiac fibrosis, Masson’s trichrome staining [43], was performed in the experimental groups. The disease group (Figure 2A(f)) stained blue color) showed higher collagen deposition as compared to the control group (Figure 2A(c)). In contrast, the antioxidant treatment revealed intact myocardium, indicating the restoration of the cellular architect. N-acetyl cysteine treatment (alloxan+N-acetyl cysteine) (Figure 2A(i)) showed more protective effects in comparison to the ascorbic acid (alloxan+ascorbic acid) (Figure 2A(r) and selenium (alloxan+selenium) treatment (Figure 2A(x)). However, the N-acetyl cysteine, ascorbic acid, and selenium alone were comparable to normal saline groups (Figure 2A(i,o,u)). Additionally, the collagen deposition intensity was measured in different experimental groups. The results show that in the disease group, collagen deposition intensity was higher than compared to control groups (Figure 2C)**.** The treatment with antioxidants prevented collagen deposition.

### 2.5. Serum Calcium Levels and Transcription Factor GATA 4 Expressions in the Diabetic Cardiac Hypertrophy Model

Increased serum calcium levels are associated with the risk of diabetic cardiac hypertrophy and aberrant calcium signaling in progressive diabetic cardiac pathological conditions [43]. Since GATA4 plays a significant role in mammalian cardiac development and serves as a mediator of cardiac hypertrophy and acts as an important regulator of calcium signaling, we studied the expression of GATA4 in diabetes-associated cardiac pathology. The effect of antioxidants on the expression of GATA4 in diabetic cardiac pathology was also studied. The serum calcium levels and mRNA expression of GATA4 were significantly upregulated in the pathological (alloxan) group in comparison to normal subjects (Figure 2D,E). In contrast, in disease groups, treatment with antioxidants restored the calcium levels and prevented oxidative damage. N-acetyl cysteine treatment (alloxan+N-acetyl cysteine) showed significant downregulation in the expression of GATA 4 as compared to ascorbic acid (alloxan+ascorbic acid) and selenium treatment (alloxan+selenium).

### 2.6. Production of Reactive Oxygen Species (ROS) Is Increased in Diabetic Cardiac Hyper-Trophy

**Figure 3:**
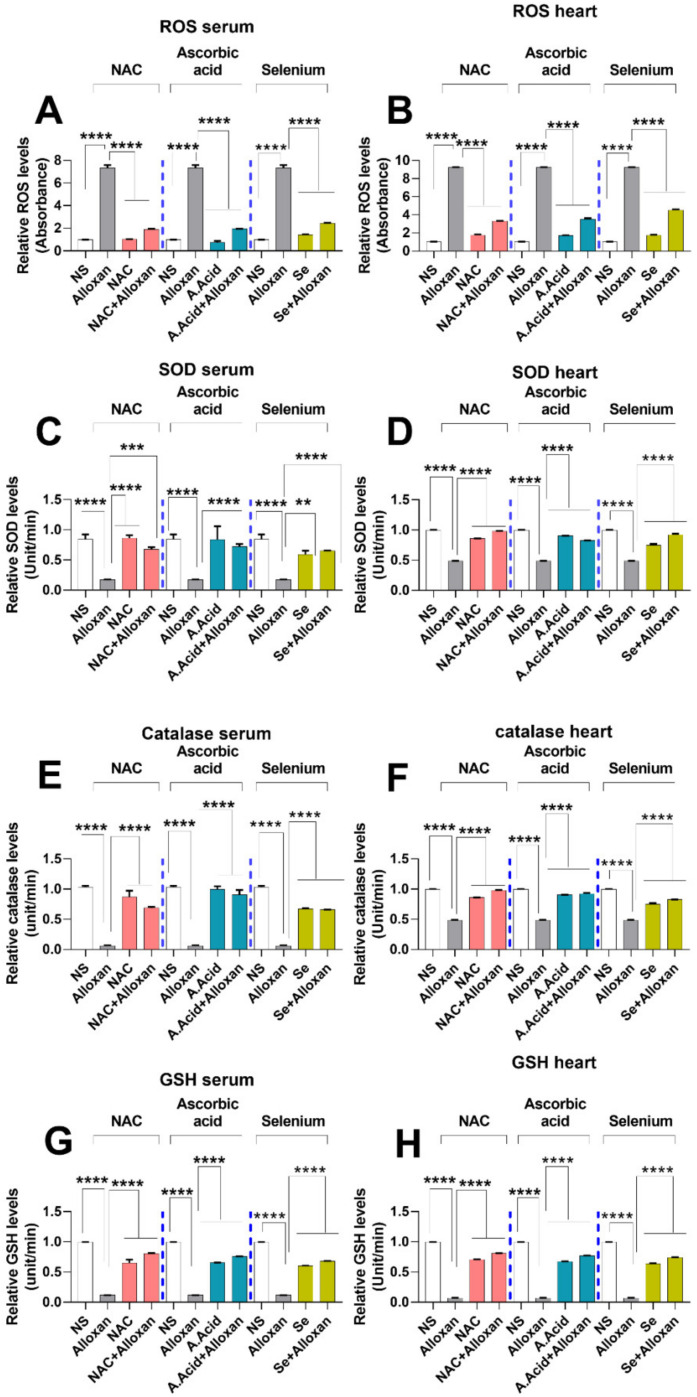
Assessment of oxidative and anti-oxidative stress profile in cardiac pathology model. (**A**) Measurement of ROS levels in serum (n = 6). (**B**) Measurement of ROS levels in heart tissue (n = 6). (**C**) Superoxide dismutase activity in serum (n = 6). (**D**) Superoxide dismutase activity in heart tissue (n = 6). (**E**) Catalase levels in serum (n = 6). **(F)** Catalase levels in tissues (n = 6). (**G**) Reduced glutathione levels in serum (n = 6). (**H**) Reduced glutathione reductase levels in heart tissues (n = 6). Data are presented as mean ± SE of n biological samples. Statistically significant differences are shown as *p*-values. Statistical analysis was performed using one-way ANOVA followed by Tukey’s test. (**): *p* < 0.01), (***): *p* < 0.001, (****): *p* < 0.0001.

An increase in oxidative stress results in an imbalance in mitochondrial dynamics such as mitochondrial fission and fusion, and is associated with cardiac pathology. The ROS production through alloxan comprises the initial step in the development and progression of the disease and can cause damage to cardiac walls [44]. NADPH oxidase is considered to be an important contributing factor in the generation of ROS in diabetic cardiac pathology [45]. Such damage can be prevented by the neutralization of ROS with antioxidants. To assess the levels of free radicals, we performed ROS quantification assay both in serum and heart tissues of the alloxan-induced cardiac hypertrophy model. The results show that the ROS levels were significantly increased in the alloxan group as compared to the normal control (Figure 3A,B).

Post ROS production analysis in the disease model, we examined whether antioxidant treatment could cause a reduction in ROS generation during diabetic-induced cardiac hypertrophy. A statistically significant decrease in ROS levels was observed when diseased subjects were treated with antioxidants, such as N-acetyl cysteine, ascorbic acid, and selenium group in serum and tissue homogenates (Figure 3A,B). The reduction in serum ROS levels was higher when treated with N-acetyl cysteine (alloxan+N-acetyl cysteine) and ascorbic acid (alloxan+ascorbic) compared to selenium (alloxan+ selenium).

### 2.7. Anti-Oxidative Defense Enzymes Are Downregulated in the Diabetic Cardiac Condition Which Is Restored by N-Acetyl Cysteine

Enzymatic antioxidants, such as superoxide dismutase (SOD), catalase (CAT), and reduced glutathione (GSH) are key components in the defense mechanisms against oxidative stress. SOD is regarded as a free radical scavenger, which can directly scavenge the reactive oxygen species and acts as first line of defense against ROS-induced damages. To examine the SOD activity, we performed SOD assay between the diseased group and those treated with antioxidants. Results show that alloxan administration impaired the standard values of SOD, CAT, and GSH profiles (Figure 3C,D), which could be due to ROS production (Figure 3A,B). The therapeutic potential of antioxidant N-acetyl cysteine, ascorbic acid, and selenium was also investigated. Our data show that the SOD activity was significantly decreased in the alloxan-treated group as compared to normal subjects both in serum and heart tissue homogenates. Specifically, the SOD activities in serum were significantly recovered (elevated) in disease groups when treated with antioxidants. The protective effects of N-acetyl cysteine treatment (alloxan+ N-acetyl cysteine) and ascorbic acid treatment (alloxan + ascorbic acid) were higher than for the selenium treatment (alloxan+ selenium). However, the levels of SOD were higher in heart tissue compared to the serum of disease groups. Of note, the serum data shows that compared to selenium, N-acetyl cysteine and ascorbic acid exhibited a better scavenging potential against ROS-induced oxidative damage.

The catalase enzyme plays an important role in the body’s defense against oxidative stress and is necessary for the conversion of hydrogen peroxide produced by superoxide dismutase activity to water [12]. Our results show that CAT levels in serum and heart tissue of the disease group were significantly reduced (Figure 3E,F). This indicates that defense against oxidative stress is hindered during diabetic cardiac hypertrophy. In contrast, CAT production was restored (elevated) with the treatment of antioxidants such as N-acetyl cysteine, ascorbic acid, and selenium (Figure 3E,F).

In addition to SOD and CAT, we also quantified glutathione levels in the serum and heart tissue of disease group relative to those treated with antioxidants. GSH has a high reduction potential and is capable of donating its reduced equivalent to other molecules such as ROS, in order to neutralize them and maintain the redox state of the cell [13]. Our data show that GSH activity was reduced in the serum and heart tissue samples of the disease group (Figure 3G,H). This indicates that the defense against oxidative stress is hindered during diabetic cardiac hypertrophy.

In contrast, the GSH level was significantly restored (increased) in the disease group when treated with antioxidants such as N-acetyl cysteine, ascorbic acid, and selenium, suggestive of their antioxidative potential. However, treatment with N-acetyl cysteine (alloxan+ N-acetyl cysteine) showed more profound effects as compared to treatment with ascorbic acid (alloxan+ ascorbic acid), and selenium (alloxan+ selenium).

### 2.8. Analysis of Lipid Peroxidation and Lipid Profile in Cardiac Pathology

**Figure 4:**
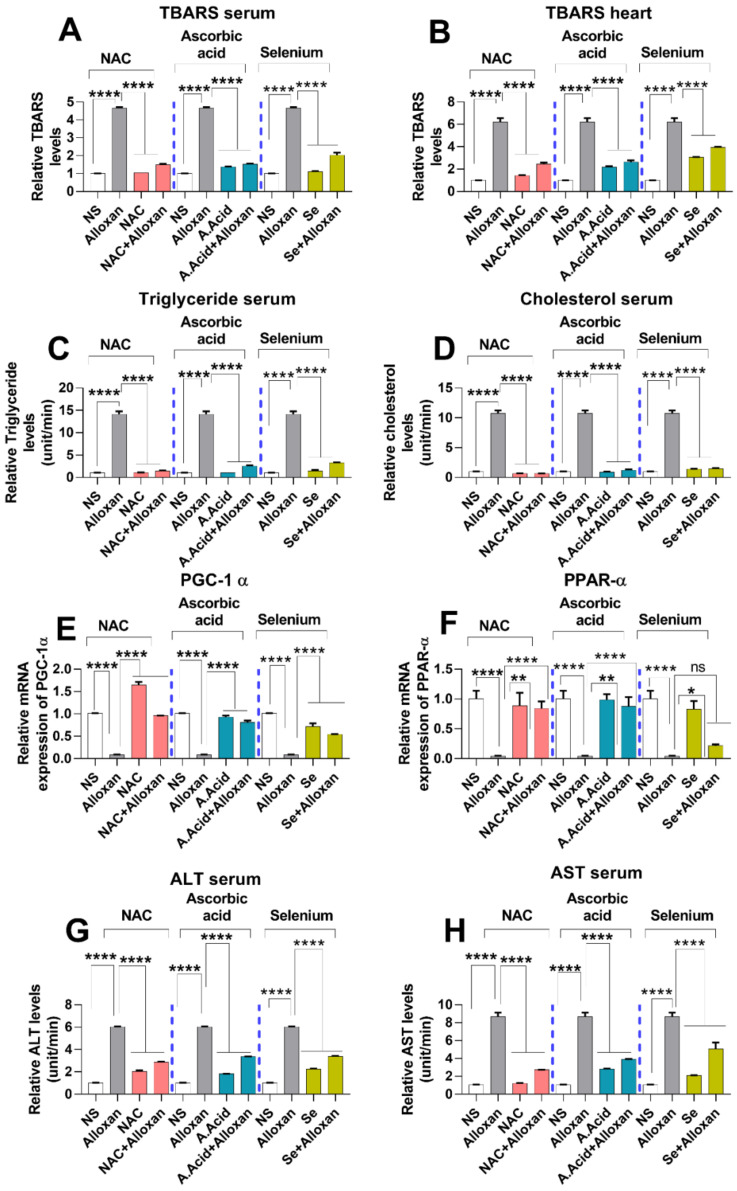
Assessment of lipid peroxidation and liver toxicity markers in cardiac pathology model. (**A**) Relative TBARS levels in serum with and without antioxidants (n = 6). (**B**) Relative TBARS levels in heart tissues (n = 6). (**C**) Relative triglyceride levels in serum (n = 6). (**D**) Relative cholesterol levels in serum (n = 6). (**E**) Relative mRNA expression of PGC-1α (n = 6) (**F**) Relative mRNA expression of PPARα (n = 6). (**G**) Relative ALT levels in serum (n = 6). (**H**) Relative AST in serum (n = 6). Data are presented as mean ± SE of n biological samples. Significant differences are shown as *p*-values. Statistical analysis was performed using one-way ANOVA followed by Tukey’s test. (*): *p* < 0.05, (**): *p* < 0.01), (****): *p* < 0.0001.

Lipids are an integral part of the body physiology, and the disturbance in lipid levels plays a central role in the development of cardiovascular diseases (CVDs) [46]. An increase in the production of free radicals initiates lipid peroxidation by damaging the membrane phospholipids and is considered to be a contributing factor in cardiac hypertrophy [47]. We performed a thiobarbituric acid reactive substance (TBARS) assay to measure the oxidative damage after alloxan administration. A significant increase in TBARS levels was observed both in serum and heart tissues of the disease group, indicating high oxidative damage to lipid integrity (Figure 4A,B).

In contrast, TBARS levels were significantly decreased in the disease group treated with antioxidants, i.e., N-acetyl cysteine, ascorbic acid, and selenium as compared to the alloxan-treated group. N-acetyl cysteine (alloxan+N-acetyl cysteine) and ascorbic-acid treatment (alloxan+ascorbic acid) conferred more protective effects against oxidative damage as compared to selenium (alloxan+selenium), both in the serum and heart tissue homogenates (Figure 4A,B).

A lipid profile is the first screening tool used to estimate deregulations in cholesterol and triglycerides. An imbalance or deregulation in the lipid profile is considered a major risk factor for cardiovascular diseases [48]. A high level of triglyceride (hypertriglyceridemia) results in an increased risk of cardiac pathologies such as cardiac ischemia, cardiac hypertrophy, and heart failure. Herein, we assessed the lipid profile in diabetes-associated cardiac hypertrophy. A significantly higher level of triglycerides was observed in the disease group. Conversely, when treated with antioxidants, a significant decrease in serum triglyceride levels was observed. N-acetyl cysteine significantly lowered the triglyceride levels in comparison to other antioxidants, suggesting its better protective potential (Figure 4C).

Additionally, cholesterol levels were also measured in the blood circulation. A high level of cholesterol leads to stroke and causes cardiovascular diseases. Results show that the serum level of circulating cholesterol was significantly high in the cardiac pathology model (Figure 4D). An elevated cholesterol level in the disease group is indicative of an altered lipid profile, which manifests as abnormal heart functioning.

In contrast, the cholesterol level was significantly decreased when treated with antioxidants, i.e., Alloxan+N-acetyl cysteine and alloxan+ascorbic acid in comparison to the allox-an+selenium treated group. N-acetyl cysteine has shown better potential in comparison to the other tested antioxidants (Figure 4D).

### 2.9. Expression of Peroxisome Proliferator-Activated Receptor-Gamma Coactivator (PGC)-1 Alpha and Peroxisome Proliferator-Activated Receptor PPARα

PGC-1α and PPARα are considered important regulators of mitochondrial fatty acid β-oxidation [49,50,51]. The qPCR data show that the mRNA expression of PGC-1α and PPARα were significantly downregulated in diabetic cardiac hypertrophy whereas treatment with antioxidants such as N-acetyl cysteine and ascorbic acid led to significantly upregulated PGC-1α and PPARα expression, in comparison to the selenium treatment group (Figure 4E,F). N-acetyl cysteine conferred a more protective response in comparison to other antioxidants.

### 2.10. Toxicity Markers Show Safer dose Administration in Diabetes-Induced Cardiac Hypertrophy Model

To measure the toxicity levels and safer dosage administration of different inducers, the liver markers were examined in the pathology model. We examined alanine aminotransferase (ALT) and aspartate aminotransferase (AST) to assess liver toxicity in all experimental groups. A prolonged disorder is responsible for the elevated levels of ALT and AST in the serum. Our results show that the levels of ALT and AST were significantly increased in the disease group in comparison to normal subjects (Figure 4G,H). In contrast, ALT and AST levels in serum of disease groups were significantly reduced when treated with antioxidants. Reduced levels of ALT and AST, upon administration of antioxidants, indicate the potential of N-acetyl cysteine, ascorbic acid, and selenium in preventing cardiac damage. N-acetyl cysteine conferred more protective effect in comparison to ascorbic acid and selenium.

### 2.11. Expression of Drp1, PUMA, and cytochrome C Is Increased in Diabetic Induced Cardiac Pathology Model

**Figure 5:**
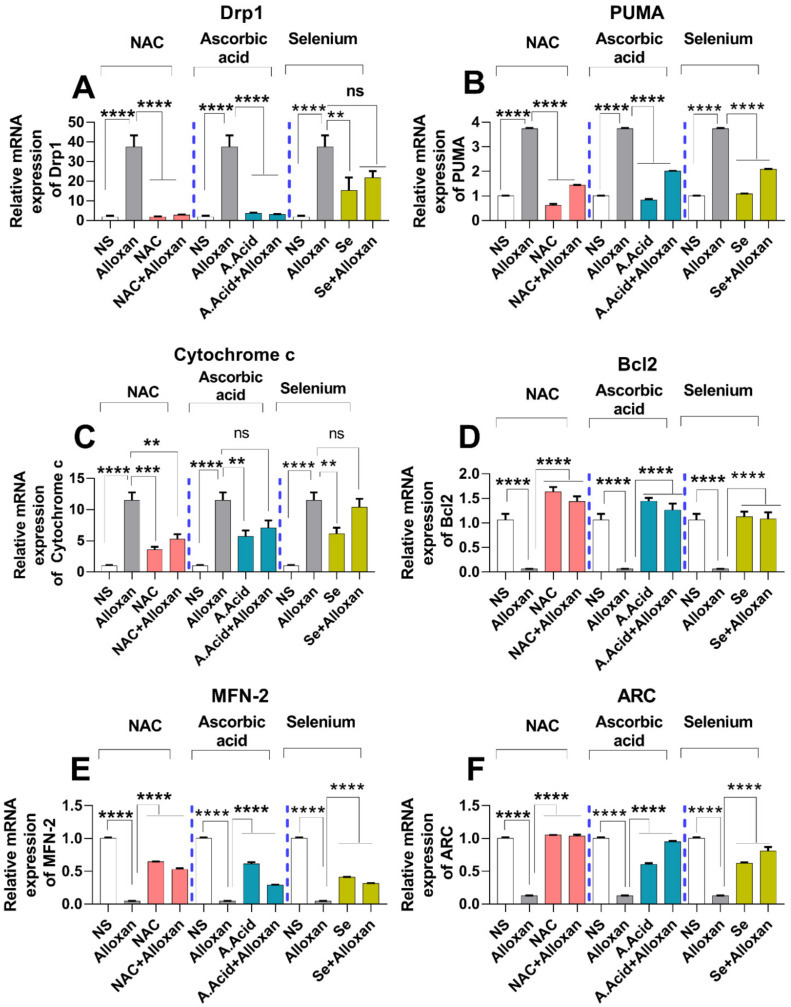
mRNA expression analysis of mitochondrial stress regulatory molecules in diabetes-induced cardiac pathology model. (**A**)Relative mRNA expression of Drp1 (n = 6). (**B**) Relative mRNA expression of PUMA (n = 6). (**C**) Relative mRNA expression of cytochrome c (n = 6). (**D**) Relative mRNA expression of anti-apoptotic genes Bcl-2 (n = 6). (**E**) Relative mRNA expression of MFN-2 (n = 6 (**F**) Relative mRNA expression of ARC (n = 6). Data are presented as mean ± SE of n biological samples (number of rats). Significant differences are shown as *p*-values. Statistical analysis was performed using one-way ANOVA followed by Tukey’s test. (**): *p* < 0.01), (***): *p* < 0.001, (****): *p* < 0.0001.

**Figure 6:**
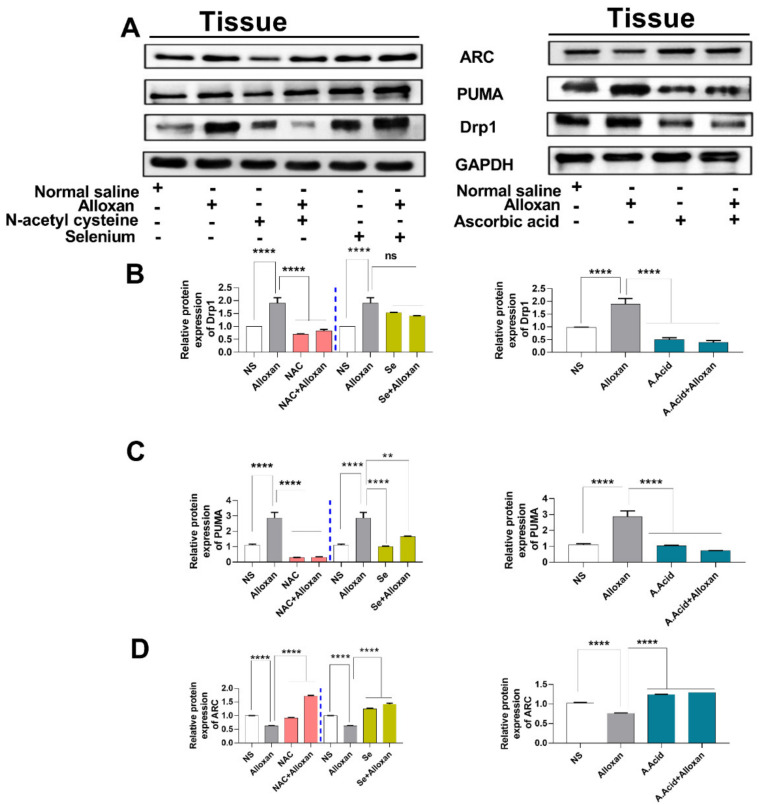
Expression analysis of mitochondrial stress regulatory proteins in diabetes-induced cardiac pathology model. (**A**) Relative protein expression of Drp1, PUMA, and ARC (GAPDH was used as loading control). (**B**) The quantitative densitometric analysis of Drp1 (n = 6) **(C)** Relative densitometric analysis of endoplasmic reticulum stress marker PUMA (n = 6) (**D**) The quantitative densitometric analysis of ARC (n = 6). Data are presented as mean ± SE of n biological samples (number of rats). Significant differences are shown as *p*-values. Statistical analysis was performed using one-way ANOVA followed by Tukey’s test. (**): *p* < 0.01), (****): *p* < 0.0001.

Mitochondrial dysfunction is considered a root cause of hyperglycemia in type 1 diabetes. Therefore, we aimed to examine the expression of Drp1 and PUMA in a diabetes-induced cardiac hypertrophy model. It is understood that Drp1 plays a major role in maintaining mitochondrial morphology and is a key regulator of mitochondrial fission [52]. The upregulation of Drp1 in various cell types occurs due to an imbalance between the mitochondrial fission and fusion, which is mainly caused by an impairment of the electron transport chain and ATP production. Our data show that the expression of Drp1 is increased in the disease group, compared to the control group Figure 5A and Figure 6A,B) both at the mRNA and protein levels. However, the treatment with N-acetyl cysteine significantly reduced the expression of Drp1 in comparison to the untreated diabetic group. The other antioxidants, i.e., ascorbic acid and selenium also showed a reduced expression of Drp1. N-acetyl cysteine exhibited more protection in comparison to ascorbic acid and selenium.

The p53 upregulated modulator of apoptosis (PUMA), also known as Bcl-2-binding component-3 (BBC3), is a pro-apoptotic gene that interacts with the anti-apoptotic Bcl-2 family members and liberates Bax and Bak [12]. It recruits the Drp1 to the outer membrane of the mitochondria and initiates a death cascade (apoptosis). Experimental data reveals that the relative mRNA and protein expression of PUMA (Figure 5B and Figure 6A,C) was significantly higher in the cardiac pathology model, indicating the induction of apoptosis. However, treatment with N-acetyl cysteine significantly downregulated the expression of apoptotic markers at the transcription (mRNA) and translation (protein) levels. N-acetyl cysteine showed higher protection in comparison to ascorbic acid and selenium.

Ascorbic acid is a standard prototype antioxidant that prevents apoptosis by inhibiting the activity of different regulators involved in apoptosis [53]. In the current study, ascorbic acid exhibited protection against ROS-mediated oxidative damage by significantly down-regulating the mRNA and protein levels of PUMA in disease groups when treated with potent antioxidants. However, selenium showed a less protective effect in the prevention of apoptotic cascade. N-acetyl cysteine shows better protective effects against apoptotic expression in comparison to ascorbic acid and selenium.

Cytochrome c expression is considered to be regulated by hypertrophic stimuli [44]. Therefore, we evaluated the relative expression of cytochrome c through real-time PCR in experimental groups. A significant increase in the cytochrome c mRNA expression was observed in the disease group. In contrast, when treated with antioxidants, a statistically significant decrease in cytochrome c mRNA level was observed in N-acetyl cysteine treatment (allox-an+N-acetyl cysteine), and in ascorbic treated groups (alloxan+ascorbic acid) compared to selenium treatment (alloxan+selenium) (Figure 5C). Collectively, the antioxidant-mediated down-regulation of Drp1, PUMA, and cytochrome c expression indicates the protective effects of antioxidants against mitochondrial oxidative stress.

### 2.12. Anti-Apoptotic Markers Bcl-2, MFN-2, and ARC Are Downregulated in Diabetic Cardiac Hypertrophy

The anti-apoptotic protein, Bcl-2, plays important role in inhibiting apoptosis [54]. We measured the relative mRNA expression of Bcl-2 in the experimental groups compared with the control groups. A significant decrease in the expression of Bcl-2 was observed in the disease group. Conversely, when treated with antioxidants, Bcl-2 expression was upregulated in all antioxidant-treated groups. N-acetyl cysteine conferred more protection against apoptotic damage in comparison to other antioxidants (Figure 5D).

The GTPase mitochondrial membrane protein, MFN-2, plays an important role in mitochondrial fusion, maintenance, and in the operation of the mitochondrial function [27]. Our data show that mRNA expression of MFN-2 was significantly downregulated in the cardiac pathology group (Figure 5E). In contrast, treatment with antioxidants conferred protective effects against mitochondrial dysfunction. The N-acetyl cysteine treatment significantly upregulated the expression of MFN-2 as compared to ascorbic acid and selenium.

Additionally, the apoptosis repressor with a caspase recruitment domain (ARC), also known as the nucleolar protein, acts as a multifunctional potent inhibitor of apoptosis. We analyzed the expression of ARC both at the transcriptional and translational levels. Our results show that the mRNA (Figure 5F) and protein expression (Figure 6A,D) of ARC was significantly downregulated in the disease group. However, treatment with N-acetyl cysteine significantly up-regulated the expression of ARC compared to ascorbic acid and selenium. A reduction in the expression of anti-apoptotic biomarkers in pathological conditions and an increased expression in disease groups, treated with antioxidants, signify the protective role of these potent antioxidants in diabetic cardiac hypertrophy. The complete Western blots are shown in Supplementary Figure S3.

### 2.13. Correlation between Diabetic, Oxidative, and Hypertrophic Parameters

A correlation analysis was performed to draw the statistical relationships between different variables in the disease group using the data from six replicates. High blood glucose levels were positively correlated with ROS-production and oxidative damage, but are negatively correlated with the expression of oxidative defense enzymes such as SOD, CAT, and GSH, and were negatively correlated with anti-apoptotic genes such as Bcl-2, and ARC. Conversely, high glucose and ROS levels were positively correlated with elevated levels of apoptotic biomarkers such as cytochrome c, DRP1, and PUMA expression along with indicators of cardiac hypertrophy such as HW/TL ratio and an increase in cell size and fibrosis. Additionally, high glucose and ROS levels were negatively correlated with regulators of mitochondrial fatty acid β-oxidation and mitochondrial maintenance such as PGC-1α, PPARα, and MFN-2. The correlation data is presented in Supplementary Table S1.

## 3. Discussion

Diabetes remains a serious threat to human health due to its high occurrence rate and serious pathophysiological conditions [45]. The maintenance of glucose homeostasis is an essential parameter for the proper functioning of metabolic pathways in the body. Malfunctioning in glucose homeostasis can lead to a condition termed hyperglycemia, which causes cardiac complications. Diabetic cardiac hypertrophy is thought to be associated with endoplasmic reticulum stress, debilitated mitochondrial signaling, disturbed calcium homeostasis, malfunctioned coronary microcirculation, and the stimulation of the renin-angiotensin-aldosterone system, among other effects [46]. Such alterations may cause multiple deleterious effects on cardiomyocytes and consequently result in heart failure [47]. One of the major hallmarks of the failing, diabetic heart is oxidative stress, and the major source of ROS production is the activity of NADPH oxidase enzymes in cardiomyocytes. NADPH oxidase-dependent mechanisms in the diabetic heart regulate many processes that promote pathological conditions including cardiac hypertrophy, interstitial fibrosis, cardiac rupture, arrhythmia, and apoptosis [48,49,50,51,52]. Alloxan monohydrate is a urea derivative (a glucose analogue), which is often found to be cytotoxic due to its generation of ROS, and is thought to demolish beta-pancreatic cells of the pancreas [40]. Alloxan administration impairs the ability of β cells to produce insulin and regulates the redox cycling of intracellular thiols, which generates ROS at multiple sites. This may lead to a pathological state of insulin-dependent diabetes or type 1-like diabetes mellitus. However, it difficult to distinguish whether the reactive oxygen species are either produced due to a necrosis of β-pancreatic cells or by persistent hyperglycemic conditions, which acts as a limitation of the alloxan-induced model [53], and thus a limitation of the current study. Persistent hyperglycemia causes the auto-oxidation of glucose and the generation of ROS. Hyperlipidemia also produces ROS through NADPH oxidase. These events cause myocardial injury by activating protein kinase C and nuclear factor-*κ*B (NF*-κ*B) [54].

**Figure 7:**
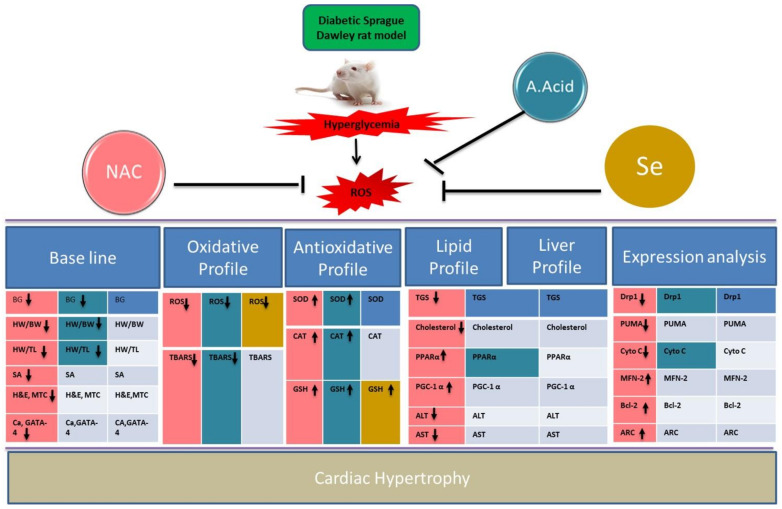
Summary representation of data in alloxan-induced cardiac hypertrophic model. Different colors show scavenging potential of antioxidants in treatment groups (Alloxan+NAC, Alloxan+A.Acid, Alloxan+Selenium). NAC showed better activity in comparison of other antioxidants. Upregulated (**↑**); Downregulated (**↓**).

The present study identifies some of the cardiac mitochondrial-stress regulatory players in a diabetes-induced cardiac malfunctioned model, with molecular deregulation and associated impaired functions (Figure 7). A successful establishment of the animal model was confirmed by glucose levels, ratios between heart weight/body weight, the heart weight/tibia length, as well as abnormal heart histology, non-uniform hypertrophied cells, an increased cell surface area, and increased fibrosis. The biochemical profiling of stress and apoptotic markers showed a significant increase in ROS production and decrease in defense-related enzymes in pathological (alloxan) groups.

Intracellular calcium ion homeostasis is a key regulator of cardiac contractility [55]. Abnormal calcium levels in diabetic cardiac hypertrophy may alter cardiac function by reducing the activity of the sarcoplasm reticulum, sarcolemmal calcium ATPase and other ion exchangers [56]. As a result of the depolarization of the cell membrane by the activation of L-type calcium channels, calcium is released from the release channels of the sarcoplasmic reticulum [57]. The increased flux of calcium is dispersed through the cytosol to reach contractile protein troponin C. Upon binding with troponin C, it leads to the sliding of thin and thick filaments, resulting in the contraction of cardiac muscles.

It has been reported that a decrease in sarcoplasmic reticulum calcium (SERCA2a) expression leads to different diabetes-associated cardiac pathologies [58]. Several studies involving diabetes models demonstrate that, in hypertrophy-driven cardiomyopathy, the rate of calcium efflux is depressed in rat myocytes. Furthermore, decreased cardiac expression of SERCA2 has been observed in both type 1 and type 2 diabetes [59,60,61]. Studies performed in diabetic patients at the time of coronary artery bypass surgery, demonstrate decreased calcium sensitivity in myofilaments, supporting the previous data obtained from animal model studies [62].

Previous data show that the altered calcium flux may change the expression of different genes at the mRNA and protein levels in diabetes mellitus [63,64,65,66]. The precise role of GATA4 has not yet been investigated in hyperglycemic-induced cardiac hypertrophy, although it has been identified as the main regulator of calcium signaling. In our findings, mRNA levels of this transcription factor were upregulated in disease (alloxan) groups, however, when disease groups were treated with antioxidants, the calcium levels were restored, and GATA4 gene expression was downregulated.

Additionally, the disease groups treated with antioxidants attenuated oxidative stress by free radicals’ scavenging mechanisms and showed a significant decrease in serum calcium levels. Diabetic cardiac hypertrophy is strictly linked with altered myocardial and substrate metabolism [67,68]. In diabetic cardiac hypertrophy, enhanced fatty acid metabolism is observed along with reduced glucose and lactate metabolism [69]. The increased fatty acid oxidation enhances the rates of oxidation in the diabetic heart, which promotes lipid accumulation in the myocardium and primes lipotoxicity [70,71,72,73]. Members of the PPARS family (PPARα, β/δ, and γ) are important regulators of cardiac metabolism, through their ligand-activated transcription factors [74]. Previous studies have shown that decreased PGC-1α and PPARα mRNA expression levels are associated with high cardiac lipid accumulation. Increased levels of triglyceride and cholesterol malfunctioning glucose transportation may progressively lead to cardiac complications [75,76]. Additionally, PGC-1α and PPARα expression were found to be down-regulated in diabetic conditions although treatment with antioxidants upregulated the expression in experimental animal model studies.

Thiobarbituric acid-reactive substances are produced as a by-product of lipid peroxidation. In the current study, a significant increase in TBARS, triglyceride and cholesterol levels were observed under diabetic conditions. Disease groups treated with antioxidants showed a significant reduction of these lipids, suggesting the protective mechanism of antioxidants against myocardial substrate metabolism.

Treatment with antioxidants N-acetyl cysteine, ascorbic acid, and selenium ameliorated oxidative stress by increasing the levels of anti-apoptotic genes and enzymes involved in antioxidant defense mechanism. Significant increases in superoxide dismutase, catalase, and GSH were observed in disease groups when treated with antioxidants, indicating protection against oxidative damage. N-acetyl cysteine exhibited better protective effects against oxidative damage as compared to ascorbic acid and selenium.

Mitochondria are the central operators of energy and the substrate metabolism. Dysfunctional mitochondrion leads to malfunctions in the respiratory chain, a reduced capacity of ATP synthesis, elevated oxidative stress, DNA damage, and an apoptosis of cardiomyocytes [77]. The expression of mitochondrial fission markers such as dynamin-related guanosine triphosphatase (GTPase) protein 1 (Drp1) and PUMA was found to be upregulated. The elevated level of cytochrome c mRNA further indicates the dysregulated mitochondrial regulation in hyperglycemic-induced cardiac hypertrophy. We investigated the protective role of antioxidants against free radical generation. N-acetyl cysteine showed more protective effects in comparison to N-acetyl cysteine and ascorbic acid. The antiapoptotic marker, Bcl-2, regulates the intrinsic apoptotic response. The expression of Bcl-2 was downregulated in the disease group. The expression of MFN-2, a marker of oxidative- stress-dependent apoptosis in cardiomyocytes, was downregulated in the disease group. However, a significant increase in Bcl-2 and MFN-2 levels was observed when treated with antioxidants. The restoration of expression of another anti-apoptotic marker, known as the apoptosis repressor with caspase recruitment domain (ARC), confirms the antioxidant potential of potent antioxidants. The N-acetyl cysteine group showed an enhanced expression of ARC as compared to the ascorbic acid group and selenium group.

## 4. Materials and Methods

### 4.1. Chemicals

Alloxan monohydrate and N-acetyl cysteine were purchased from Sigma-Aldrich (Darmstadt, Germany). Ascorbic acid was obtained from Scharlau, and Selenium was obtained from DAEJUNG (Korea). Phenylmethanesulfonyl Fluoride (PMSF) was purchased from (Roth Germany) CAS no: 329-98-6.

### 4.2. Study Design

Sprague Dawley rats were obtained from the Animal House of the Department of Biological Sciences, Quaid-i-Azam University, Islamabad, Pakistan. Rats of both sexes were divided into 8 experimental groups and each group which comprised of 6 rats that were randomly divided at a ratio of 1:1 in each experimental group. The body weight of all animals ranged between (120 g–180 g) [78,79] and they ranged from 4–6 weeks old (Figure 1A). All animals were fed on normal feed and water. To prevent hypoglycemic shocks, the diabetic group was fed on water containing a 5% glucose solution for 18 h [80,81,82].

Normal saline group: In this group, normal saline (0.9%) 500 μL was administered subcutaneously for consecutive 11 days.

Diabetic group: Disease was induced in this group by administering a single intraperitoneal injection of alloxan (120 mg/Kg), following 24 h of fasting (Alloxan was dissolved in normal saline) [80].

Ascorbic acid group: Ascorbic Acid (80 mg/Kg) was subcutaneously administered to this group for 11 consecutive days.

Alloxan + Ascorbic acid group: Diabetic rats were treated subcutaneously with ascorbic acid (80 mg/Kg) for 11 consecutive days, followed by the induction of diabetes with a single alloxan dose [42,80].

N-acetyl cysteine group: NAC (50 mg/Kg) was administered subcutaneously to this group for 11 days consecutively.

Alloxan + N-acetyl cysteine group: After inducing diabetes as in the disease group, this group was treated subcutaneously with NAC (50 mg/Kg) for 11 consecutive days [24,80].

Selenium group: 0.6 mg/kg selenium dissolved in 2.5% DMSO was administered orally for 11 consecutive days.

Alloxan + Selenium group: A single injection of alloxan as 120 mg/kg was followed by selenium dosing for 11 consecutive days [29,31,80].

The 24-h dosage interval was followed for all antioxidants. All animal- handling procedures were according to the standard protocols documented in the “Guide for the Care and Use of 1L2 laboratory Animals (National Institute of Health, Islamabad, Pakistan) as per the considerations of the U.K., Animals (Scientific Procedures) Act, 1986 and approved by the ethical committee of Quaid-i-Azam University Islamabad, Pakistan (Ethical approval number: #BEC-FBS-QAU2017-67). Rats were anesthetized and their glucose level was measured. Hearts were obtained from all the animals and washed with chilled 0.9% normal saline and distilled water to remove blood. Organs were weighed using an electric weighing machine and stored at −20 °C. Organs were fixed in 10% formalin and processed for histopathological analysis. For RNA extraction, a portion of heart tissue was treated with TRIzol and stored at −70 °C. All the spectrophotometry experiments were carried out using 8453 UV–Visible spectrophotometer (Agilent, Santa Clara, CA, USA).

### 4.3. Measurement of Blood Glucose Level

Blood glucose levels were measured by the strip method using FreeStyle Optium glucometer (Abbot, Alameda, CA, USA) according to the manufacturer’s instructions. The results were recorded in units of mg/dl in triplicates for each sample.

### 4.4. Serum Separation from Blood

The blood samples from all experimental models were collected in gel tubes. Blood was centrifuged at 6000 rpm for 10 min and the clear upper layer was pipetted into Eppendorf tubes. The serum samples were stored at −20 °C for further molecular analysis.

### 4.5. Tissue Homogenization

Heart tissues (100mg) were homogenized using an electric homogenizer according to the previously described protocol [83]. After homogenization, centrifugation was performed at 13,000 rpm at 4 °C for 10 min. The upper clear-enriched extracted phase was obtained and was used in a biochemical analysis and Western blot analysis.

### 4.6. Reactive Oxygen Species (ROS) Assay

For measurements of the amount of ROS in experimental rat models, the previously described protocols were followed. Absorbance was measured at 505 nm using 8453 UV–Visible spectrophotometer (Agilent, Santa Clara, CA, USA). Three readings were taken for each sample after an interval of 15 s and their average was calculated [84]. For the measurement of ROS, a H_2_O_2_ standard calibration curve was plotted, and levels of ROS were measured.

### 4.7. Thiobarbituric Acid Reactive Substances (TBARS) Assay

The protocol of Buege and Austin was followed for measurement of TBARS activity [85]. Optical density was measured at 532nm, using spectrophotometer.The following formula was used to calculate Lipid peroxidation products as Nm/mg TBARS (Nm/mg protein) = O.D × Total volume× Sample volume × 1.56 × 105 × mg protein/mL (1.56 × 105 = Molar Extinction Coefficient).

### 4.8. Superoxide Dismutase (SOD) Assay

The activity of SOD in serum and tissue homogenates was evaluated using the assay of the previously described protocol [86]. Absorbance was measured at 560nm and three readings were taken within 1 min. The following formula was used to calculate the percentage inhibition of NBT:

Inhibition of NBT = (A blank – A sample)/(A blank) × 100(1)

The amount of enzyme causing 50% inhibition in NBT reduction rate is defined as “one unit of SOD activity”. SOD activity was measured on a unit/minute scale.

### 4.9. Catalase Activity (CAT) Assay

A catalase assay was also performed, following the previously optimized protocol [24]. Absorbance was measured three times at 240nm.Without a sample, the mixture was considered blank. The one-unit activity of catalase is described as an “absorbance change of 0.01 as unit per minute”.

### 4.10. Reduced Glutathione (GSH) Assay

For an evaluation of the glutathione reductase activity in experimental rats, GSH activity was evaluated by previously described protocols. Absorbance was recorded at 412 nm [87].

### 4.11. Lipid Profiling

The lipids, including triglyceride and cholesterol, were profiled through AMP Diagnostic kit, Austria. All experimental procedures were carried out according to the manufacturer’s instructions [42].

### 4.12. Calcium Test

Serum calcium levels were also measured by using the AMP diagnostic kit, Austria according to the manufacturer’s instructions.

### 4.13. Liver Profiling

#### 4.13.1. Alanine aminotransferase (ALT) assay

ALT was performed using the AMP diagnostic kit protocol. The ALT-enzyme level was checked in serum. Absorbance was obtained at a wavelength of 340nm. The following formula was used for the measurement of enzymatic activity [42].

∆A/min × 3333 = ALT activity (unit/litter) at 37 °C(2)

#### 4.13.2. Aspartate Aminotransferase (AST) Assay

AST enzyme levels were detected in experimental models by using the AMP kit method. Absorbance was obtained at a 340 nm wavelength. The following formula was used for calculation [42].

∆A/min × 3333 = AST activity (unit/litter) at 37 °C(3)

### 4.14. Western Blot Analysis

The relative protein expression of biomarkers was analyzed by immunoblotting. Firstly, for the quantification of proteins, a Bradford assay was performed. Samples were subjected to denaturation (boiled at 100 °C for 10 min) and then separated on 12% SDS-PAGE. Samples were then transferred to the nitrocellulose membranes, following the protocol described in [24]. After blocking with non-fat skimmed milk, the membrane was treated with primary antibodies (Drp1:sc-271583, PUMA:sc-374223, ARC sc-374177; Santa Cruz Biotechnology Texas, USA, GAPDH; K00027; Solarbio, China) at [1:5000] dilutions for overnight incubation at 4 °C, followed by secondary antibody [goat anti-mouse antibody ab-97020 IgG-AP; Abcam, UK 1: 2000] dilution. For the detection of signals, a 1 step TM NBT/BCIP cat no.34042 Thermo-Fisher Scientific substrate was used according to the manufacturer’s instructions, to subsequently visualize blots. The images were recorded and further quantified by Image J software.

### 4.15. Histological Analysis

For the morphological evaluation, the formalin-fixed, paraffin-embedded specimens were cut into 3 µm sections followed by Hematoxylin Eosin as described previously [24]. Additionally, Masson’s trichrome staining was also performed according to published protocols [88]. The obtained sections were then observed under polarized light and bright-field microscope (DIALUX 20 EB, Olympus, Tokyo, Japan) at 40X and 4× magnifications, respectively. Cell surface areas were measured by SPOT CamW 4.0. Software [24]. Collagen intensity was measured using a collagen recipe, through MIPAR ^TM.^_2017_ software [89].

### 4.16. Real-Time PCR Analysis

RNA extraction was conducted using the previously described protocols [24]. Total RNA was extracted with TRIzol reagent (Invitrogen) and quantified by UV/Vis Nano-Drop TM Spectrophotometer (NanoDrop, V3.7, Thermo-Fisher Scientific, USA) in ng/μL units. RNA was treated with DNase 1 (Thermo-Fisher Scientific, USA) and absorption was observe at 260 nm (A260) and 280 nm (A280). cDNA synthesis was carried out from RNA using Thermofisher scientific Revert Aid First Strand cDNA Synthesis Kit, according to the instructional protocol in a thermocycler (T100 Bio-Rad Thermocycler, USA). Specific gene primers were designed from the NCBI database and Ensemble genome Browser. Primers were purchased from IDT and BJI (China) and were optimized using a conventional PCR. The list of primers is presented in Table 1. Real-time PCR reaction mix was prepared using SYBER green master mix (Thermo-fisher scientific) kit. RT PCR reaction was run in the MyGo Pro instrument. The expression of target genes was quantified, and data were normalized against GAPDH as an internal control.

### 4.17. Statistical Analysis

Data were analyzed using Prism software (GraphPad Software, USA). To evaluate significant differences among different experimental groups, the statistical tests, including multiple comparison tests ANOVA followed by Tukey’s test, were applied. All data were presented as a mean ± SE of available samples number. *P* values of less than 0.05 were considered statistically significant. A correlation analysis was performed to identify the statistical relationships between biological variables in pathological groups using the data for all six replicates. The values for the Pearson correlation coefficients (or Pearson’s *r*) were calculated between all variables using GraphPad Prism.

## 5. Conclusions

Our data identify numerous dysregulations in the diabetic-associated cardiac pathology model, including the abnormal expression of mitochondrial stress-associated molecules, and hypertrophy of the heart-tissue environment—both reflected locally and from the secretion of macromolecules in the circulation. Additionally, N-acetyl cysteine was found to be a potential protective agent, and may be used to ameliorate diabetes-linked cardiac pathology. An upregulation of apoptotic and cardiac hypertrophic biomarkers may provide further insight into pathology-related signaling cascades, whereby the downregulation of apoptotic biomarkers may explain the improved scavenging potential of antioxidants. Since N-acetyl cysteine, a specific standard antioxidant, demonstrated better scavenging potential against oxidative damage, it could be further exploited for enhanced therapeutic strategies in treating diabetic heart conditions.

## Figures and Tables

**Table 1:** List of gene primers for real-time PCR.

	Target	Gene ID	Primer	Sequence
Rattus norvegicus	PPARα	25747	FP	5′-ATGAACAAAGACGGGATGC-3′
			RP	5′-TCAAACTTGGGTTCCATGAT-3′
	GATA-4	54254	FP	5′-CCCATGGGTCCTCCATC-3′
			RP	5′-GGGGGTGCTGATTACG-3′
	DRP1	114114	FP	5′-GATGCCATAGTTGAAGTGGTGAC-3′
			RP	5′-CCACAAGCATCAGCAAAGTCTGG-3′
	PUMA	317673	FP	5′-ACGACCTCAACGCACAGTACGA-3′
			RP	5′-CCTAATTGGGCTCCATCTCGGG-3′
	MFN-2	64476	FP	5-′TCAAGACCGTGAACCAGC-3′
			RP	5′-AGAAGTGGACACTTGGAGTTG-3′
	GAPDH	24383	FP	5′-TTCAACAGCAACTCCCATT -3′
			RP	5′-CACCACCCTGTTGCTGTA-3′
	ARC	85383	FP	5-TGCCAGGAGCTGCTACGCTGT-3
			RP	5-TGGGCATGGAGGGTCATAGCTG-3

FP, Forward Primer; RP, Reverse Primer.

## Data Availability

All the available data is contained in the manuscript. Some figures are provided in the Supplementary Data.

## Supplementary Materials

The following are available online, Figure S1: comparison of initial and final body weight change in experimental groups, Figure S2: comparison of initial and final blood glucose levels, Figure S3: Representative images of western blots, Table S1: Correlation between diabetic, oxidative and hypertrophic or fibrotic parameters

## Sample Availability

Samples of all the compounds that are used in this study will be available from the authors I.M. (Iram Mushtaq) and I.M. (Iram Murtaza).

## Abbreviations

ROS: Reactive Oxygen Species
TBARS: Thiobarbituric Acid Reactive Substances
SOD: Superoxide Dismutase assay
CAT: Catalase
ALT: Alanine Aminotransferase Assay
AST: Aspartate Aminotransferase Assay
Drp1: Drp1 (dynamin-related guanosine triphosphatase (GTPase) protein 1)
PUMA: p53 upregulated modulator of apoptosis
GATA-4: GATA binding protein 4
MFN-2: Mitofusin-2
ARC: Apoptosis repressor with caspase recruitment domain
NAC: N-acetyl Cysteine
PPARα: peroxisome proliferator-activated receptors (PPARs) alpha
FIS1: Mitochondrial fission 1 protein
PDK4: Pyruvate dehydrogenase lipoamide kinase isozyme 4
ACC: acetyl-Co-A carboxylase
CPT1: carnitine palmitoyltransferase I
SERCA2a: Sarcoplasmic reticulum calcium pumps
AMPK: AMP-dependent protein kinase
AGEs: advanced glycation end products
BG: Blood glucose
SA: surface area
TGS: Triglyceride

## References

[1] Ojiako O.A., Chikezie P.C., Ogbuji A.C. (2015). Blood glucose level and lipid profile of alloxan-induced hyperglycemic rats treated with single and combinatorial herbal formulations. J. Tradit. Complement. Med..

[2] Feingold K.R., Brinton E.A., Grunfeld C. (2020). The effect of endocrine disorders on lipids and lipoproteins. Endotext.

[3] Ritchie R.H., Abel E.D. (2020). Basic Mechanisms of Diabetic Heart Disease. Circ. Res..

[4] Jia G., Whaley-Connell A., Sowers J.R. (2017). Diabetic cardiomyopathy: A hyperglycaemia- and insulin-resistance-induced heart disease. Diabetologia.

[5] Barzilay J.I., Kronmal R.A., Gottdiener J.S., Smith N.L., Burke G.L., Tracy R., Savage P.J., Carlson M. (2004). The association of fasting glucose levels with congestive heart failure in diabetic adults ≥65 years: The Cardiovascular Health Study. J. Am. Coll. Cardiol..

[6] Mushtaq S., Ali T., Altaf F., Abdullah M., Murtaza I. (2015). Stress-responsive factor regulation in patients suffering from type 2 diabetes and myocardial infarction. Turk. J. Med. Sci..

[7] Lee T.-W., Bai K.-J., Chao T.-F., Kao Y.-H., Chen Y.-J. (2017). PPARs modulate cardiac metabolism and mitochondrial function in diabetes. J. Biomed. Sci..

[8] Thirupathi A., De Souza C.T. (2017). Biochemistry, Multi-regulatory network of ROS: The interconnection of ROS, PGC-1 alpha, and AMPK-SIRT1 during exercise. J. Physiol. Biochem..

[9] Boudina S., Abel E.D. (2007). Diabetic Cardiomyopathy Revisited. Circulation.

[10] Mushtaq S., Ali T., Gul M., Javed Q., Emanueli C., Murtaza I. (2015). Insulin over expression induces heart abnormalities via reactive oxygen species regulation, might be step towards cardiac hypertrophy. Cell. Mol. Boil..

[11] Ali T., Waheed H., Shaheen F., Mahmud M., Javed Q., Murtaza I. (2015). Increased endogenous serotonin level in diabetic conditions may lead to cardiac valvulopathy via reactive oxygen species regulation. Biologia.

[12] Jan M.I., Khan R.A., Ali T., Bilal M., Bo L., Sajid A., Malik A., Urehman N., Waseem N., Nawab J. (2017). Interplay of mitochondria apoptosis regulatory factors and microRNAs in valvular heart disease. Arch. Biochem. Biophys..

[13] Ribas V., García-Ruiz C., Fernández-Checa J.C. (2014). Glutathione and mitochondria. Front. Pharmacol..

[14] Zhang Y., Murugesan P., Huang K., Cai H. (2019). NADPH oxidases and oxidase crosstalk in cardiovascular diseases: Novel therapeutic targets. Nat. Rev. Cardiol..

[15] Birben E., Sahiner U.M., Sackesen C., Erzurum S., Kalayci O. (2012). Oxidative stress and antioxidant defense. World Allergy Organ J..

[16] Dludla P.V., Dias S., Obonye N., Johnson R., Louw J., Nkambule B.B. (2018). A Systematic Review on the Protective Effect of N-Acetyl Cysteine Against Diabetes-Associated Cardiovascular Complications. Am. J. Cardiovasc. Drugs.

[17] Wang T., Qiao S., Lei S., Liu Y., Ng K.F.J., Xu A., Lam K.S.L., Irwin M.G., Xia Z. (2011). N-Acetylcysteine and Allopurinol Synergistically Enhance Cardiac Adiponectin Content and Reduce Myocardial Reperfusion Injury in Diabetic Rats. PLoS ONE.

[18] Okazaki T., Otani H., Shimazu T., Yoshioka K., Fujita M., Iwasaka T. (2011). Ascorbic acid and N-acetyl cysteine prevent uncoupling of nitric oxide synthase and increase tolerance to ischemia/reperfusion injury in diabetic rat heart. Free. Radic. Res..

[19] Kumar S., Sitasawad S.L. (2009). N-acetylcysteine prevents glucose/glucose oxidase-induced oxidative stress, mitochondrial damage and apoptosis in H9c2 cells. Life Sci..

[20] Xia Z., Guo Z., Nagareddy P., Yuen V., Yeung E., McNeill J.H. (2006). Antioxidant N-acetylcysteine restores myocardial Mn-SOD activity and attenuates myocardial dysfunction in diabetic rats. Eur. J. Pharmacol..

[21] Johnson R., Sangweni N.F., Mabhida S.E., Dludla P.V., Mabasa L., Riedel S., Chapman C., Mosa R.A., Kappo A.P., Louw J. (2019). An in vitro study on the combination effect of metformin and N-acetyl cysteine against hyperglycae-mia-induced cardiac damage. Nutrients.

[22] Xia Z., Kuo K.-H., Nagareddy P., Wang F., Guo Z., Guo T., Jiang J., McNeill J.H. (2007). N-acetylcysteine attenuates PKCβ2overexpression and myocardial hypertrophy in streptozotocin-induced diabetic rats. Cardiovasc. Res..

[23] Fiordaliso F., Bianchi R., Staszewsky L., Cuccovillo I., Doni M., Laragione T., Salio M., Savino C., Melucci S., Santangelo F. (2004). Antioxidant treatment attenuates hyperglycemia-induced cardiomyocyte death in rats. J. Mol. Cell. Cardiol..

[24] Ali T., Mushtaq I., Maryam S., Farhan A., Saba K., Jan M.I., Sultan A., Anees M., Duygu B., Hamera S. (2018). Interplay of N acetyl cysteine and melatonin in regulating oxidative stress-induced cardiac hypertrophic factors and microRNAs. Arch. Biochem. Biophys..

[25] Mushtaq S., Ali T., Javed Q., Tabassum S., Murtaza I. (2015). N-acetyl cysteine inhibits endothelin-1-induced ROS de-pendent cardiac hypertrophy through superoxide dismutase regulation. Cell J..

[26] Scalzo R.L., Bauer T.A., Harrall K., Moreau K., Ozemek C., Herlache L., McMillin S., Huebschmann A.G., Dorosz J., Reusch J.E.B. (2018). Acute vitamin C improves cardiac function, not exercise capacity, in adults with type 2 diabetes. Diabetol. Metab. Syndr..

[27] Aluwong T., Ayo J.O., Kpukple A., Oladipo O.O. (2016). Amelioration of Hyperglycaemia, Oxidative Stress and Dyslipidaemia in Alloxan-Induced Diabetic Wistar Rats Treated with Probiotic and Vitamin C. Nutrients.

[28] Mehdi Y., Hornick J.-L., Istasse L., Dufrasne I. (2013). Selenium in the Environment, Metabolism and Involvement in Body Functions. Molecules.

[29] He Y., Chen S., Liu Z., Cheng C., Li H., Wang M. (2014). Toxicity of selenium nanoparticles in male Sprague–Dawley rats at supranutritional and nonlethal levels. Life Sci..

[30] Żarczyńska K., Sobiech P., Tobolski D., Mee J.F., Illek J. (2021). Effect of a single, oral administration of selenitetriglycerides, at two dose rates, on blood selenium status and haematological and biochemical parameters in Holstein-Friesian calves. Ir. Vet. J..

[31] Takada H., Hirooka T., Hatano T., Hamada Y., Yamamoto M. (1992). Inhibition of 7, 12-dimethylbenz [a] anthracene-induced lipid peroxidation and mammary tumor development in rats by vitamin E in conjunction with selenium. Nutr. Cancer.

[32] Hall A.R., Burke N., Dongworth R.K., Hausenloy D.J. (2014). Mitochondrial fusion and fission proteins: Novel therapeutic targets for combating cardiovascular disease. Br. J. Pharmacol..

[33] Rosca M.G., Hoppel C.L. (2010). Mitochondria in heart failure. Cardiovasc. Res..

[34] Pasula D.J., Shi R., Shih A.Z., Luciani D.S., Shi R. (2018). Anti-Apoptotic Bcl-xL Limits Mitochondrial Dysregulation in β-cells during Prolonged Exposure to High Glucose. Can. J. Diabetes.

[35] Kroemer G., Dallaporta B., Resche-Rigon M. (1998). The mitochondrial death/life regulator in apoptosis and necrosis. Annu. Rev. Physiol..

[36] Loo J.F., Lau P., Ho H., Kong S. (2013). An aptamer-based bio-barcode assay with isothermal recombinase polymerase amplification for cytochrome-c detection and anti-cancer drug screening. Talanta.

[37] Waterhouse N.J., Trapani J.A. (2003). A new quantitative assay for cytochrome c release in apoptotic cells. Cell Death Differ..

[38] Chandra D., Liu J.-W., Tang D., Canela N., Rodriguez-Vilarrupla A., Estanyol J.M., Dıaz C., Pujol M.J., Agell N., Bachs O. (2002). Early Mitochondrial Activation and Cytochrome c Up-regulation during Apoptosis. J. Biol. Chem..

[39] Giunti S., Bruno G., Veglio M., Gruden G., Webb D.J., Livingstone S., Chaturvedi N., Fuller J.H., Perin P.C., The Eurodiab Iddm Complications Study Group (2005). Electrocardiographic Left Ventricular Hypertrophy in Type 1 Diabetes: Prevalence and relation to coronary heart disease and cardiovascular risk factors: The Eurodiab IDDM Complications Study. Diabetes Care.

[40] Novoa U., Arauna D., Moran M., Nuñez M., Zagmutt S., Saldivia S., Valdes C., Villaseñor J., Zambrano C.G., Gonzalez D.R. (2017). High-intensity exercise reduces cardiac fibrosis and hypertrophy but does not restore the nitroso-redox imbalance in diabetic cardiomyopathy. Oxid. Med. Cell. Longev..

[41] Whitman V., Schuler H.G., Neely J.R. (1979). Effect of alloxan-induced diabetes on the hypertrophic response of rat heart. J. Mol. Cell. Cardiol..

[42] Mushtaq I., Mushtaq I., Akhter Z., Murtaza I., Qamar S., Ayub S., Mirza B., Butt T.M., Janjua N.K., Shah F.U. (2019). Engineering electroactive and biocompatible tetra(aniline)-based terpolymers with tunable intrinsic antioxidant properties in vivo. Mater. Sci. Eng. C.

[43] Bisping E., Ikeda S., Kong S.W., Tarnavski O., Bodyak N., McMullen J.R., Rajagopal S., Son J.K., Ma Q., Springer Z. (2006). Gata4 is required for maintenance of postnatal cardiac function and protection from pressure overload-induced heart failure. Proc. Natl. Acad. Sci. USA.

[44] Xia Y., Buja L.M., McMillin J.B. (1998). Activation of the cytochrome c gene by electrical stimulation in neonatal rat cardiac myocytes: Role of NRF-1 and c-Jun. J. Biol. Chem..

[45] Jia G., DeMarco V., Sowers J.R. (2015). Insulin resistance and hyperinsulinaemia in diabetic cardiomyopathy. Nat. Rev. Endocrinol..

[46] Goldberg I.J., Trent C.M., Schulze P.C. (2012). Lipid Metabolism and Toxicity in the Heart. Cell Metab..

[47] Sciarretta S., Volpe M., Sadoshima J. (2014). Mammalian Target of Rapamycin Signaling in Cardiac Physiology and Disease. Circ. Res..

[48] Zhang M., Perino A., Ghigo A., Hirsch E., Shah A.M. (2013). NADPH Oxidases in Heart Failure: Poachers or Gamekeepers?. Antioxid. Redox Signal..

[49] Proud C.G. (2004). Ras, PI3-kinase and mTOR signaling in cardiac hypertrophy. Cardiovasc. Res..

[50] Malhowski A.J., Hira H., Bashiruddin S., Warburton R., Goto J., Robert B., Kwiatkowski D.J., Finlay G.A. (2011). Smooth muscle protein-22-mediated deletion of Tsc1 results in cardiac hypertrophy that is mTORC1-mediated and reversed by rapamycin. Hum. Mol. Genet..

[51] Inoki K., Li Y., Xu T., Guan K.-L. (2003). Rheb GTPase is a direct target of TSC2 GAP activity and regulates mTOR signaling. Genes Dev..

[52] Cesario D.A., Brar R., Shivkumar K. (2006). Alterations in Ion Channel Physiology in Diabetic Cardiomyopathy. Endocrinol. Metab. Clin. N. Am..

[53] Ighodaro O.M., Adeosun A.M., Akinloye O.A. (2017). Alloxan-induced diabetes, a common model for evaluating the glycemic-control potential of therapeutic compounds and plants extracts in experimental studies. Medicina.

[54] Al-Rasheed N.M., Al-Rasheed N.M., Hasan I., Al-Amin M.A., Al-Ajmi H.N., Mohamad R.A., Mahmoud A.M. (2017). Simvastatin Ameliorates Diabetic Cardiomyopathy by Attenuating Oxidative Stress and Inflammation in Rats. Oxid. Med. Cell. Longev..

[55] Zhao X.-Y., Hu S.-J., Li J., Mou Y., Chen B.-P., Xia Q. (2006). Decreased cardiac sarcoplasmic reticulum Ca2+-ATPase activity contributes to cardiac dysfunction in streptozotocin-induced diabetic rats. J. Physiol. Biochem..

[56] Endoh M. (2006). Signal Transduction and Ca2+ Signaling in Intact Myocardium. J. Pharmacol. Sci..

[57] Choi K.M., Zhong Y., Hoit B.D., Grupp I.L., Hahn H., Dilly K.W., Guatimosim S., Lederer W.J., Matlib M.A. (2002). Defective intracellular Ca2+ signaling contributes to cardiomyopathy in Type 1 diabetic rats. Am. J. Physiol. Circ. Physiol..

[58] Pereira L., Matthes J., Schuster I., Valdivia H.H., Herzig S., Richard S., Gómez A.M. (2006). Mechanisms of [Ca2+] i Transient Decrease in Cardiomyopathy of db/db Type 2 Diabetic Mice. Diabetes.

[59] Hattori Y., Matsuda N., Kimura J., Ishitani T., Tamada A., Gando S., Kemmotsu O., Kanno M. (2000). Diminished function and expression of the cardiac Na+-Ca2+exchanger in diabetic rats: Implication in Ca2+overload. J. Physiol..

[60] Kashihara H., Shi Z.Q., Yu J.Z., McNeill J.H., Tibbits G.F. (1999). Effects of diabetes and hyper-tension on myocardial Na+-Ca2+ exchange. Can. J. Physiol. Pharmacol..

[61] Belke D.D., Swanson E.A., Dillmann W.H. (2004). Decreased Sarcoplasmic Reticulum Activity and Contractility in Diabetic db/db Mouse Heart. Diabetes.

[62] Jweied E.E., McKinney R.D., Walker L.A., Brodsky I., Geha A.S., Massad M.G., Buttrick P.M., De Tombe P.P. (2005). Depressed cardiac myofilament function in human diabetes mellitus. Am. J. Physiol. Circ. Physiol..

[63] Trost S.U., Belke D.D., Bluhm W.F., Meyer M., Swanson E., Dillmann W.H. (2002). Overexpression of the Sarcoplasmic Reticulum Ca2+-ATPase Improves Myocardial Contractility in Diabetic Cardiomyopathy. Diabetes.

[64] Simpson D.L., Brooks C.L. (1999). Tailoring the structural integrity process to meet the challenges of aging aircraft. Int. J. Fatigue.

[65] Sen L., Cui G., Fonarow G., Laks H. (2000). Differences in mechanisms of SR dysfunction in ischemic vs. idiopathic dilated cardiomyopathy. Am. J. Physiol. Circ. Physiol..

[66] Lopaschuk G.D. (2002). Metabolic Abnormalities in the Diabetic Heart. Heart Fail. Rev..

[67] Taegtmeyer H., McNulty P., Young M.E. (2002). Adaptation and maladaptation of the heart in diabetes: Part I: General concepts. Circulation.

[68] Stanley W.C., Lopaschuk G.D., McCormack J.G. (1997). Regulation of energy substrate metabolism in the diabetic heart. Cardiovasc. Res..

[69] Carley A., Severson D.L. (2005). Fatty acid metabolism is enhanced in type 2 diabetic hearts. Biochim. Biophys. Acta (BBA) Mol. Cell Biol. Lipids.

[70] Frazier-Wood A., Ordovas J., Straka R., Hixson J., Borecki I., Tiwari H., Arnett D. (2012). The PPAR alpha gene is associated with triglyceride, low-density cholesterol and inflammation marker response to fenofibrate intervention: The GOLDN study. Pharmacogenomics J..

[71] Lee T.-I., Kao Y.-H., Chen Y.-C., Huang J.-H., Hsiao F.-C., Chen Y.-J. (2013). Peroxisome prolifera-tor-activated receptors modulate cardiac dysfunction in diabetic cardiomyopathy. Diabetes Res. Clin. Pract..

[72] Sharma S., Adrogue J.V., Golfman L., Uray I.P., Lemm J., Youker K., Noon G.P., Frazier O.H., Taegtmeyer H. (2004). Intramyocardial lipid accumulation in the failing human heart resembles the lipotoxic rat heart. FASEB J..

[73] Szczepaniak L.S., Dobbins R.L., Metzger G., Sartoni-D’Ambrosia G., Arbique D., Vongpatanasin W., Unger R., Victor R.G. (2003). Myocardial triglycerides and systolic function in humans: In vivo evaluation by localized proton spectroscopy and cardiac imaging. Magn. Reson. Med..

[74] Zhou Y.-T., Grayburn P., Karim A., Shimabukuro M., Higa M., Baetens D., Orci L., Unger R.H. (2000). Lipotoxic heart disease in obese rats: Implications for human obesity. Proc. Natl. Acad. Sci. USA.

[75] Puddu G.M., Cravero E., Arnone G., Muscari A., Puddu P. (2005). Molecular aspects of atherogenesis: New insights and unsolved questions. J. Biomed. Sci..

[76] Finck B.N., Chinetti G., Staels B. (2008). PPARs/RXRs in Cardiovascular Physiology and Disease. PPAR Res..

[77] Chistiakov D.A., Shkurat T.P., Melnichenko A.A., Grechko A.V., Orekhov A.N. (2018). The role of mitochondrial dysfunction in cardiovascular disease: A brief review. Ann. Med..

[78] Boye A., Barku V.Y.A., Acheampong D.O., Ofori E.G. (2021). Abrus precatorius Leaf Extract Reverses Allox-an/Nicotinamide-Induced Diabetes Mellitus in Rats through Hormonal (Insulin, GLP-1, and Glucagon) and Enzymatic (α-Amylase/α-Glucosidase) Modulation. BioMed Res. Int..

[79] Naz I., Khan M.R., Zai J.A., Batool R., Maryam S., Majid M. (2021). Indigofera linifolia ameliorated CCl4 induced endoplasmic reticulum stress in liver of rat. J. Ethnopharmacol..

[80] Ali T., Ishtiaq A., Mushtaq I., Ayaz N., Jan M.I., Khan W., Khan U., Murtaza I. (2021). Mentha longifolia Alleviates Exog-enous Serotonin-Induced Diabetic Hypoglycemia and Relieves Renal Toxicity via ROS Regulation. Plant Foods Hum. Nutr..

[81] Ebuehi O., Ajuluchukwu A., Afolabi O., Akinwande A. (2010). Oxidative stress in alloxan-induced diabetes in female and male rats. Adv. Med Dent. Sci..

[82] Surya D., Vijayakumar R.S., Nalini N. (2005). Oxidative stress and the role of cumin (*Cuminum cyminum* Linn.) in allox-an-induced diabetic rats. J. Herbs Spices Med. Plants.

[83] Ma H., Shieh K.J.J. (2006). Western blotting method. J. Am. Sci..

[84] Hayashi I., Morishita Y., Imai K., Nakamura M., Nakachi K., Hayashi T. (2007). High-throughput spectrophotometric assay of reactive oxygen species in serum. Mutat. Res. Toxicol. Environ. Mutagen..

[85] Buege J.A., Aust S.D. (1978). Microsomal Lipid Peroxidation. Methods in Enzymology.

[86] Jevremović S., Petrić M., Živković S., Trifunović M., Subotić A. (2010). Superoxide dismutase activity and isoen-zyme profiles in bulbs of snake’s head fritillary in response to cold treatment. Arch. Biol. Sci..

[87] Rahman I., Kode A., Biswas S.K. (2006). Assay for quantitative determination of glutathione and glutathione disulfide levels using enzymatic recycling method. Nat. Protoc..

[88] Schipke J., Brandenberger C., Rajces A., Manninger M., Alogna A., Post H., Mühlfeld C. (2017). Assessment of cardiac fibrosis: A morphometric method comparison for collagen quantification. J. Appl. Physiol..

[89] Moudgil R., Samra G., Ko K.A., Vu H.T., Thomas T.N., Luo W., Chang J., Reddy A.K., Fujiwara K., Abe J.-I. (2020). Topoisomerase 2B Decrease Results in Diastolic Dysfunction via p53 and Akt: A Novel Pathway. Front. Cardiovasc. Med..

