# Supplementary material for: N-Acetyl Cysteine, Selenium, and Ascorbic Acid Rescue Diabetic Cardiac Hypertrophy via Mitochondrial-Associated Redox Regulators
Source: Molecules. 2021 Nov 30;26(23):7285. doi: 10.3390/molecules26237285 (PMC8659237; doi:10.3390/molecules26237285)
Supplement: Supplementary file 1 [file molecules-26-07285-s001.zip › molecules-1458218-supplementary.pdf]

molecules

Supplementary data

Iram Mushtaq [1], Zainab Bashir [1], Mehvish Sarwar [1], Maria Arshad [1], Ayesha Ishtiaq [1], Wajiha Khan [2], Uzma Khan [3], Sobia Tabassum [4], Tahir Ali [1], Tahzeeb Fatima [5], Hadi Valadi [5], Muhammad Nawaz [5,*] and Iram Murtaza [1,*]

1 Signal transduction Laboratory, Department of Biochemistry, Faculty of Biological Sciences, Quaid-i-Azam University, Islamabad 45320, Pakistan. irammushtaq@bs.qau.edu.pk; zainabbashir23@gmail.com; mehwishmalik609@gmail.com, m.arshad@bs.qau.edu.pk, tahirali.bch@gmail.com; ayesha@bs.qau.edu.pk; irambch@qau.edu.pk

2 Department of Biotechnology, COMSATS University Islamabad, Abbottabad Campus, Abbotabad 22060, Pakistan. wajihak@cuiatd.edu.pk

3 Faculty of Biological Sciences, Hazara University, Mansehra 21040, Pakistan. uzmaqau2003@yahoo.com

4 Department of Bioinformatics and Biotechnology, IIUI, Islamabad, 44000, Pakistan. sobia.tabasum@iiu.edu.pk.

5 Department of Rheumatology and Inflammation Research, Institute of Medicine, Sahlgrenska Academy, University of Gothenburg, 413 46 Gothenburg, Sweden tahzeeb.fatima@gu.se ; hadi.valadi@gu.se ; muhammad.nawaz@gu.se

* Correspondence: muhammad.nawaz@gu.se (M.N.); irambch@qau.edu.pk (I.M.)

Citation: Mushtaq, I.; Bashir, Z.; Sarwar, M.; Arshad, M.; Ishtiaq, A.; Khan, W.; Khan, U.; Tabassum, S.; Ali, T.; Fatima, T.; et al. N-acetyl Cysteine, Selenium, and Ascorbic Acid Rescue Diabetic Cardiac Hypertrophy via Mitochondrial-Associated Redox Regulators. *Molecules* **2021**, *26*, 7285. https://doi.org/10.3390/molecules26237285

Academic Editor: Francesca Giampieri

Received: 26 October 2021
Accepted: 29 November 2021
Published: 30 November 2021

Publisher's Note: MDPI stays neutral with regard to jurisdictional claims in published maps and institutional affiliations.

Copyright: © 2021 by the authors. Submitted for possible open access publication under the terms and conditions of the Creative Commons Attribution (CC BY) license (http://creativecommons.org/licenses/by/4.0/).

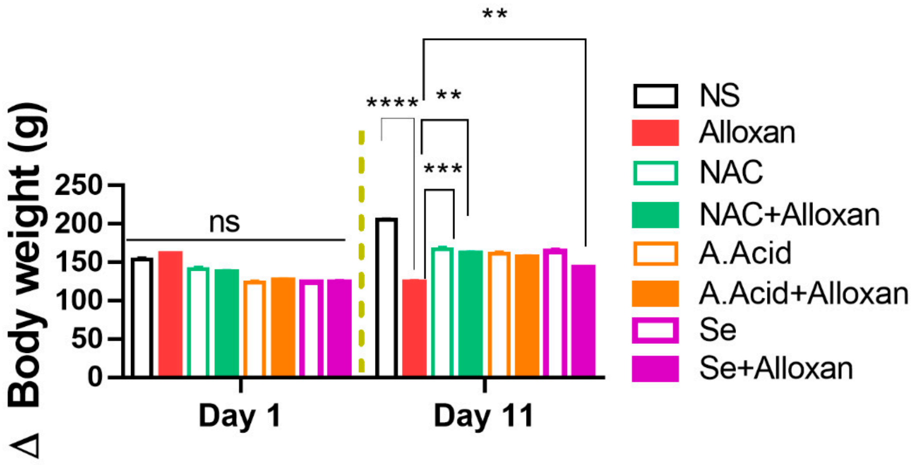

Supplementary Figure S1: comparison of initial and final body weight change in experimental groups. Statistical analysis was performed using Two-way ANOVA followed by Tukey's test. (**): $p < 0.01$), (***): $p < 0.001$, (****): $p < 0.0001$

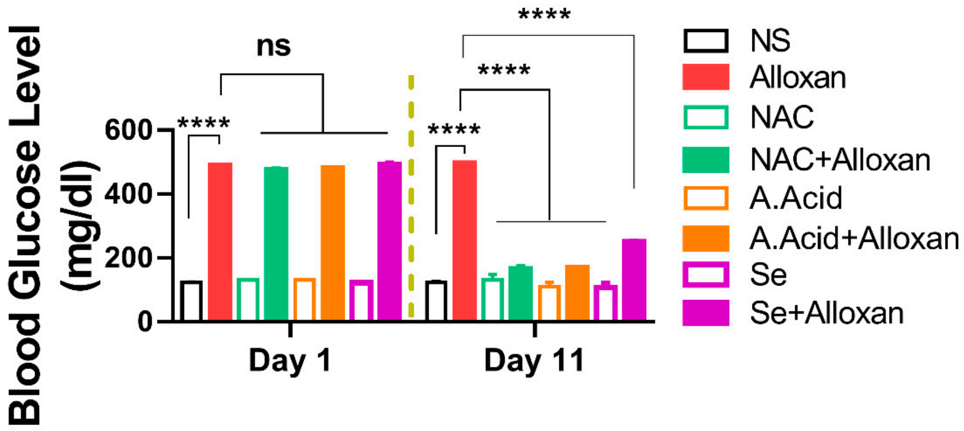

Supplementary Figure S2: comparison of initial and final blood glucose levels. Statistical analysis was performed using two-way ANOVA followed by Tukey's test. (****): $p < 0.0001$

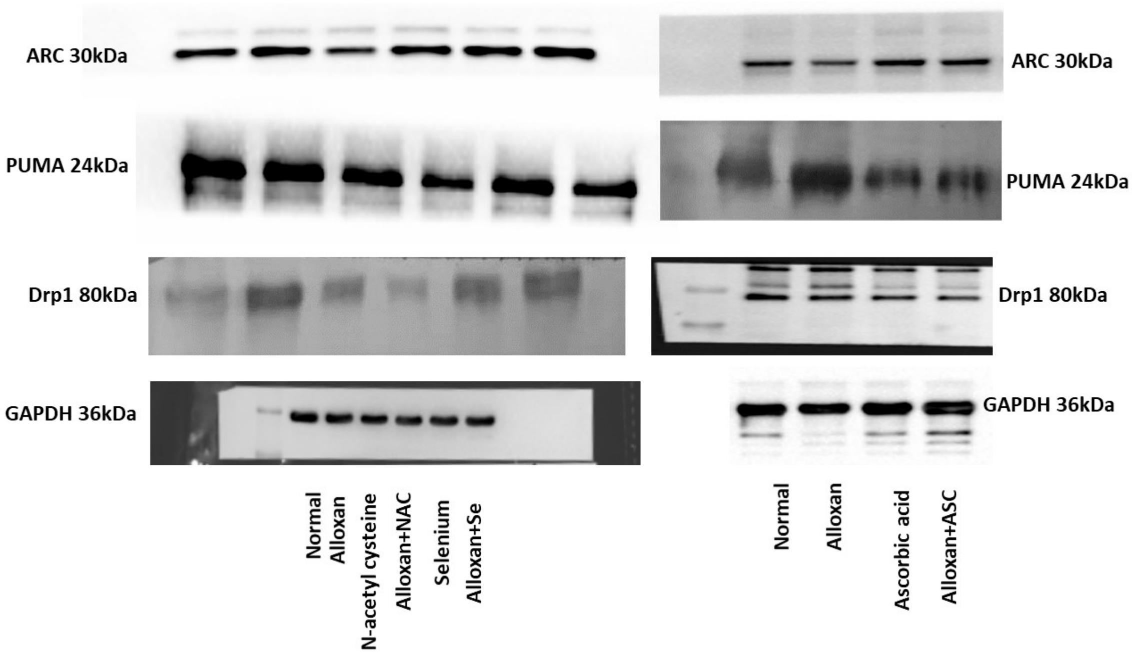

Supplementary Figure S3: Representative images of western blots

molecules

MDPI

Supplementary Table S1: Correlation between diabetic, oxidative and hypertrophic or fibrotic parameters

	ROS (S)	ROS (T)	Glucose	SOD (S)	SOD (T)	CAT (S)	CAT (T)	GSH (S)	GSH (T)	mRNA (Drp1)	mRNA (PUMA)	mRNA (ARC)	Drp1 (Prot.)	PUMA (Prot.)	ARC (Prot.)	mRNA (MFN-2)	mRNA (PPARα)	HW/BW	HW/TL	Cell SA	Fibrosis	mRNA (PGC-1a)	mRNA (Bcl2)	mRNA (Cyt C)
ROS (S)																								
ROS (T)	0.99**																							
Glucose	1.00	0.99																						
SOD (S)	-0.96	-0.99	-0.96																					
SOD (T)	-1.00	-0.97	-1.00*	0.93																				
CAT (S)	-0.97	-1.00*	-0.97	1.00*	0.95																			
CAT (T)	-1.00*	-1.00*	-1.00*	0.98	0.99	0.99																		
GSH (S)	-1.00*	-1.00*	-1.00	0.98	0.98	0.99	1.00**																	
GSH (T)	-1.00*	-1.00*	-1.00*	0.98	0.99	0.99	1.00**	1.00																
mRNA (Drp1)	0.99	0.97	1.00	-0.92	-1.00	-0.94	-0.98	-0.98	-0.98															
mRNA (PUMA)	1.00**	0.99	1.00*	-0.97	-0.99	-0.98	-1.00*	-1.00	-1.00	0.99														
mRNA(ARC)	-0.99	-0.95	-0.99	0.90	1.00*	0.92	0.97	0.97	0.97	-1.00	-0.98													
Drp1 (Prot.)	0.98	0.94	0.98	-0.88	-0.99	-0.90	-0.96	-0.96	-0.96	0.99	0.97	-1.00*												
PUMA(P)	0.90	0.84	0.91	-0.75	-0.94	-0.78	-0.87	-0.87	-0.88	0.95	0.90	-0.96	0.98											
ARC(P)	-0.93	-0.97	-0.92	1.00	0.89	0.99	0.95	0.95	0.95	-0.88	-0.93	0.85	-0.82	-0.68										
mRNA(MFN-2)	-0.93	-0.97	-0.92	1.00	0.89	0.99	0.95	0.95	0.95	-0.88	-0.93	0.85	-0.82	-0.68	1.00*									
mRNA(PPARα)	-1.00**	-0.99	-1.00**	0.96	0.99	0.98	1.00*	1.00**	1.00*	-0.99	-1.00**	0.98	-0.97	-0.90	0.93	0.93								
HW/BW	0.99	1.00**	0.99	-0.98	-0.98	-0.99	-1.00*	-1.00*	-1.00*	0.98	1.00*	-0.96	0.95	0.85	-0.96	-0.96	-1.00*							
HW/TL	1.00**	0.99	1.00*	-0.95	-1.00*	-0.97	-1.00*	-0.99	-1.00*	1.00*	1.00*	-0.99	0.98	0.91	-0.92	-0.92	-1.00*	0.99						
Cell SA	1.00*	0.98	1.00*	-0.95	-1.00*	-0.97	-0.99	-0.99	-1.00	1.00*	1.00*	-0.99	0.98	0.92	-0.91	-0.91	-1.00*	0.99	1.00**					
Fibrosis	1.00*	0.98	1.00*	-0.94	-1.00*	-0.96	-0.99	-0.99	-0.99	1.00*	1.00*	-0.99	0.99	0.93	-0.91	-0.91	-1.00	0.99	1.00*	1.00**				
mRNA(PGC1a)	-0.99	-0.97	-1.00	0.92	1.00**	0.94	0.98	0.98	0.99	-1.00**	-0.99	1.00*	-0.99	-0.95	0.88	0.88	0.99	-0.98	-1.00*	-1.00*	-1.00*			
mRNA(BcL2)	-0.90	-0.83	-0.91	0.74	0.94	0.78	0.87	0.86	0.87	-0.94	-0.89	0.96	-0.97	-1.00*	0.67	0.67	0.89	-0.85	-0.91	-0.92	-0.92	0.94		
mRNA (Cyt C)	0.96	0.99	0.96	-1.00**	-0.93*	-1.00	-0.98	-0.98	-0.98	0.92	0.96	-0.90	0.88	0.75	-1.00	-1.00	-0.96	0.98	0.95	0.95	0.94	-0.92	-0.74	

Supplementary data

The correlation scale for Pearson correlation coefficients (Pearson's *r*) varies from -1 to +1 for all variables.

+1 or closer to +1 indicate strongly positively correlated, whereas -1 or closer to -1 indicate strongly negatively correlated.

S= serum; T= tissue, p= protein; SA= surface area; CytC = cytochrome c

Correlational analysis, significance (*p*) at

< 0.01**

< 0.05*
